# A GIPR antagonist conjugated to GLP-1 analogues promotes weight loss with improved metabolic parameters in preclinical and phase 1 settings

**DOI:** 10.1038/s42255-023-00966-w

**Published:** 2024-02-05

**Authors:** Murielle M. Véniant, Shu-Chen Lu, Larissa Atangan, Renee Komorowski, Shanaka Stanislaus, Yuan Cheng, Bin Wu, James R. Falsey, Todd Hager, Veena A. Thomas, Malhar Ambhaikar, Lucie Sharpsten, Yineng Zhu, Vamsi Kurra, Rohini Jeswani, Rajneet K. Oberoi, Jane R. Parnes, Narimon Honarpour, Joel Neutel, Jennifer L. Strande

**Affiliations:** 1 Amgen Research, Department of Cardiometabolic Disorders, Thousand Oaks, CA USA; 2 Amgen Research, Department of Therapeutic Discovery, Thousand Oaks, CA USA; 3 Amgen Research, Department of Translational Safety & Bioanalytical Sciences, Thousand Oaks, CA USA; 4 Amgen Research, Department of Pharmacokinetics and Drug Metabolism, South San Francisco, CA USA; 5 Pre-pivotal Drug Substance Technologies, Amgen, Thousand Oaks, CA USA; 6 Amgen Early Development, Amgen, Thousand Oaks, CA USA; 7 Orange County Research Center, Tustin, CA USA

**Keywords:** Obesity, Metabolic syndrome, Metabolism, Translational research, Clinical trial design

## Abstract

Obesity is a major public health crisis. Multi-specific peptides have emerged as promising therapeutic strategies for clinical weight loss. Glucagon-like peptide-1 (GLP-1) and glucose-dependent insulinotropic polypeptide (GIP) are endogenous incretins that regulate weight through their receptors (R). AMG 133 (maridebart cafraglutide) is a bispecific molecule engineered by conjugating a fully human monoclonal anti-human GIPR antagonist antibody to two GLP-1 analogue agonist peptides using amino acid linkers. Here, we confirm the GIPR antagonist and GLP-1R agonist activities in cell-based systems and report the ability of AMG 133 to reduce body weight and improve metabolic markers in male obese mice and cynomolgus monkeys. In a phase 1, randomized, double-blind, placebo-controlled clinical study in participants with obesity (NCT04478708), AMG 133 had an acceptable safety and tolerability profile along with pronounced dose-dependent weight loss. In the multiple ascending dose cohorts, weight loss was maintained for up to 150 days after the last dose. These findings support continued clinical evaluation of AMG 133.

## Abstract (web summary)

Veniant et al. report here on a GIPR antagonist conjugated to GLP-1 analogues that reduces body weight and improves metabolic markers in preclinical and phase 1 clinical settings.

## Main

Obesity is a heterogeneous, chronic condition that has grown into an extremely prevalent public health problem^1^ and is one of the greatest threats to human health and well-being in the 21st century. The morbidity associated with obesity has exerted a tremendous burden on patients and the healthcare system globally^2^, accounting for an estimated $173 billion in medical expenditures in the United States in 2019 (ref. ^3^). Effective and safe obesity therapeutics are needed^4,5^. The advancement of incretin-based therapeutics has resulted in a sea change in the management of type 2 diabetes, with the most recently developed agents promoting notable reductions in weight as well^6–8^. These large reductions in weight by current incretin therapeutics have translated into improvements in cardiovascular health. In a recent clinical study, individuals with heart failure and preserved ejection fraction who received weekly semaglutide injections (2.4 mg) for 52 weeks experienced substantially improved symptoms and physical function compared to the placebo group^9^. Furthermore, the SELECT trial showed a 20% reduction in major adverse cardiovascular event risk in patients with obesity and without diabetes who lost weight with semaglutide treatment^10^. Therapeutics with enhanced efficacy, improved pharmacokinetics (PK) allowing less-frequent dosing and good tolerability remain in high demand to address the unmet medical need of patients with obesity.

GLP-1 and GIP are gut-derived incretin hormones that augment glucose-stimulated insulin secretion and have been shown to have important roles in weight regulation. In addition to the incretin effects, GLP-1 promotes satiety^11^ and GIP promotes lipid storage and metabolic regulation^12–15^. Several GLP-1R agonists (GLP-1RA) have been approved to treat type 2 diabetes and have demonstrated benefits in managing obesity^16^. While GLP-1RA continue to be successful^17^, combinatorial approaches are being developed due to the hypothesis that additional pathway engagement will generate greater weight loss, durability of weight loss and improved tolerability^7^. Unimolecular multi-specific agonist peptides targeting multiple incretin pathways have emerged as promising therapeutic strategies for weight loss^7,8,18–26^.

Genome-wide association studies (GWAS) in humans show that the *GIPR* locus contributes to body weight (BW) regulation and GIPR knockout mice are protected from diet-induced obesity (DIO)^27–32^. Pharmacological inhibition of GIPR with anti-GIPR antibodies prevented BW gain in DIO mice and obese cynomolgus monkeys^33^. Furthermore, GIPR antagonism in combination with GLP-1R agonism synergistically reduced BW in DIO mice and obese cynomolgus monkeys^33^, suggesting the potential for a GIPR/GLP-1R bispecific molecule for improving the efficacy of obesity treatment. To test this hypothesis, a series of GIPR antagonist antibodies (GIPR-Ab) conjugated to GLP-1 peptides were created and investigated^34^. These bispecific molecules that antagonize GIPR and agonize GLP-1R pathways led to decreases in BW and improvements in metabolic parameters in obese mice and monkeys^34^.

AMG 133 (now known as maridebart cafraglutide) is an optimized GIPR/GLP-1R bispecific molecule engineered by conjugating a fully human monoclonal anti-human GIPR-Ab with two GLP-1 analogue agonist peptides using amino acid linkers. Here we report its design, preclinical development and clinical proof-of-concept study to evaluate the safety, tolerability, PK and pharmacodynamics (PD) of AMG 133.

## Results

### AMG 133 structure and in vitro potency

**Fig. 1:**
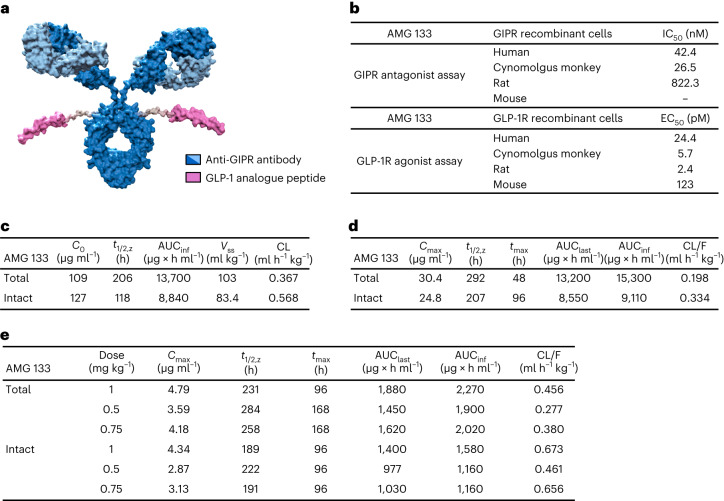
AMG 133 exhibited GIPR antagonist and GLP-1R agonist activities in vitro and extended PK profiles. **a**, Structure of AMG 133. The GLP-1 analogue peptide sequence is H[Aib]EGTFTSDYSSYLEEQAAKEFIAWLVKGGG(GGGGS)3 K(BrAc){CONH2}. **b**, In vitro potency of AMG 133. **c**–**e**, PK properties after a single dose of 5 mg kg^−1^ i.v. administration of AMG 133 to CD-1 mice (**c**), a single dose of 3 mg kg^−1^ s.c. administration of AMG 133 to female cynomolgus monkeys (**d**) and s.c. administration of AMG 133 to obese male cynomolgus monkeys (**e**). AUC, area under the curve; AUC_inf_, AUC zero to infinity; *V*_ss_, volume in steady state; CL, clearance; F, fraction absorbed; EC_50_, half maximal effective concentration; GIPR, glucose-dependent insulinotropic polypeptide receptor; GLP-1R, glucagon-like peptide-1 receptor; IC_50_, half maximal inhibitory concentration; IV, intravenous; PK, pharmacokinetic; SC, subcutaneous; t_max_, time of maximum observed concentration.

**Extended Data Fig. 1:**
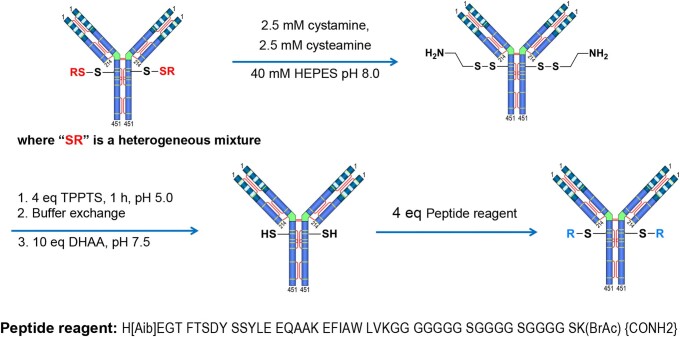
Conjugation of GLP-1 peptide analog to GIPR antibody. AMG 133 chemical structure.

AMG 133 is an antibody–peptide conjugate. A fully human monoclonal GIPR-Ab is specifically conjugated at the E384C positions to a GLP-1 agonist peptide analogue using a linker, resulting in two GLP-1 agonist peptides per antibody molecule (Fig. 1a). The average molecular weight of AMG 133 is 153,514 Da and the peptide sequence including the linker is H[Aib]EGTFTSDYSSYLEEQAAKEFIAWLVKGGG(GGGGS)3K(BrAc)^20^. The characteristics of the compound are displayed in Extended Data Fig. 1.

**Extended Data Fig. 2:**
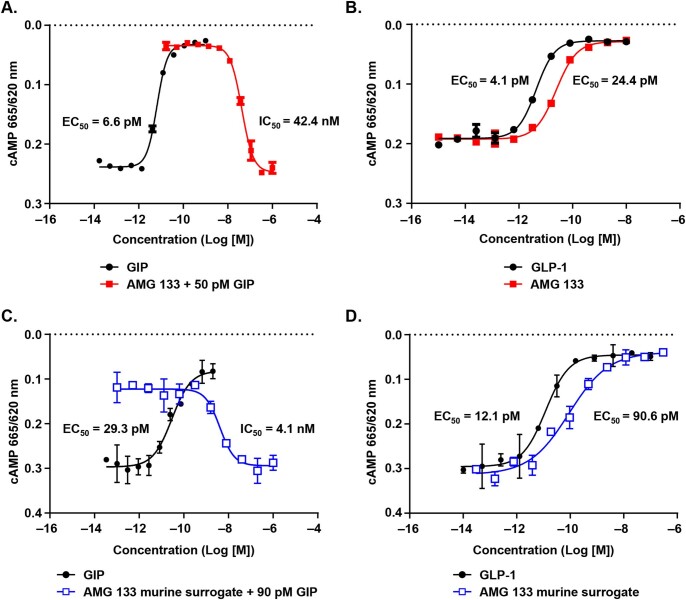
In vitro functional activity of AMG 133 or AMG 133 murine surrogate. (**a–d**) Representative dose-response curves of cAMP assays in cells expressing human GIPR (A), human GLP-1R (B), mouse GIPR (C), or mouse GLP-1R (D). 50 pM or 90 pM GIP was used in the antagonist mode (A&C) of the assay. Data are represented as mean ± SEM of n = 2 replicates per treatment. Source data

In cell-based functional assays, AMG 133 shows antagonist activity against human, cynomolgus monkey and rat GIPR (Fig. 1b). Human embryonic kidney (HEK) 293T cells recombinantly expressing human GIPR or cynomolgus monkey GIPR and Chinese hamster ovary (CHO) cells expressing rat or mouse GIPR were used to measure cyclic adenosine monophosphate (cAMP) accumulation. Native GIP produces full agonism of the human GIPR (half maximal effective concentration (EC_50_) = 6.6 pM) and AMG 133 fully antagonizes native GIP (Extended Data Fig. 2a). Native GLP-1 and AMG 133 both induce complete agonism of human GLP-1R, with respective EC_50_ values of 4.1 and 24.4 pM (Extended Data Fig. 2b). AMG 133 functionally inhibits GIP signalling through human and cynomolgus monkey GIPR with similar potency (half maximal inhibitory concentration (IC_50_) of 42.4 nM and 26.5 nM, respectively), whereas it has more than 20-fold lower potency for inhibition of GIP signalling through rat GIPR (IC_50_ = 822 nM) and did not fully antagonize mouse GIPR with concentrations of up to 3 µM. Therefore, an anti-mouse GIPR surrogate antibody was used to conjugate with the GLP-1 analogue peptide to generate an AMG 133 murine surrogate to assess its effect in pharmacology studies in mice. GIPR antagonist activity of the murine surrogate was evaluated in CHO-AM1D cells recombinantly expressing mouse GIPR. The endogenous ligand GIP results in full agonism of the mouse GIPR, and the AMG 133 murine surrogate fully antagonized native GIP (IC_50_ = 4.1 nM) (Extended Data Fig. 2c).

Agonist activities of AMG 133 were evaluated by measuring cAMP levels in CHO-K1 cells recombinantly expressing human or mouse GLP-1R or CHO-AM1D cells expressing cynomolgus monkey GLP-1R (Fig. 1b). AMG 133 results in full agonism of human GLP-1R (EC_50_ = 24.4 pM) and activated cynomolgus monkey and mouse GLP-1R (EC_50_ of 5.7 and 123 pM, respectively). The AMG 133 murine surrogate stimulates cAMP production in CHO-K1 cells expressing mouse GLP-1R with EC_50_ values of 90.6 pM (Extended Data Fig. 2d).

### AMG 133 PK profile in mice and obese cynomolgus monkeys

AMG 133 exhibits persistent PK properties after a single intravenous (i.v.) injection of 5 mg kg^−1^ in mice (Fig. 1c). The PK profiles of intact AMG 133 (GIPR-Ab with at least one attached GLP-1 analogue peptide) and total AMG 133 (GIPR-Ab with or without GLP-1 analogue peptides) follow a biphasic clearance pattern and demonstrate similar initial concentrations between assays with a gradual divergence over the 2-week study period. Intact AMG 133 shows faster mean clearance than total AMG 133 (0.568 versus 0.367 ml h^−1^ kg^−1^). Total AMG 133 has a longer mean terminal half-life (*t*_1/2,z_) of 206 h compared to intact AMG 133, which has a mean *t*_1/2,z_ of 118 h. The PK profile after a single subcutaneous (s.c.) injection of 3 mg kg^−1^ AMG 133 in female cynomolgus monkeys is shown in Fig. 1d. The PK profiles of intact and total AMG 133 demonstrate similar initial concentrations between assays with a gradual divergence over the 5-week study period. Consistent with CD-1 mouse PK data, intact AMG 133 has a shorter mean *t*_1/2,z_ compared to total AMG 133 (207 h versus 292 h). The mean plasma PK parameters after s.c. administration of AMG 133 to obese male cynomolgus monkeys are shown in Fig. 1e. In the considered dose range of 0.5 to 1 mg kg^−1^, AMG 133 demonstrates a long half-life ranging from 189 to 222 h (intact AMG 133 assay) and from 231 to 284 h (total AMG 133 assay). Plasma AMG 133 exposure as assessed by maximum observed concentration (*C*_max_) and area under the concentration–time curve (AUC) from time 0 to the time of the last measured concentration (AUC_last_) increases with increasing dose. Intact and total AMG 133 exposures more closely approximate each other in the initial phase of the study than in the terminal phase (Fig. 1e).

### AMG 133 reduces BW in DIO mice and obese cynomolgus monkeys

**Fig. 2:**
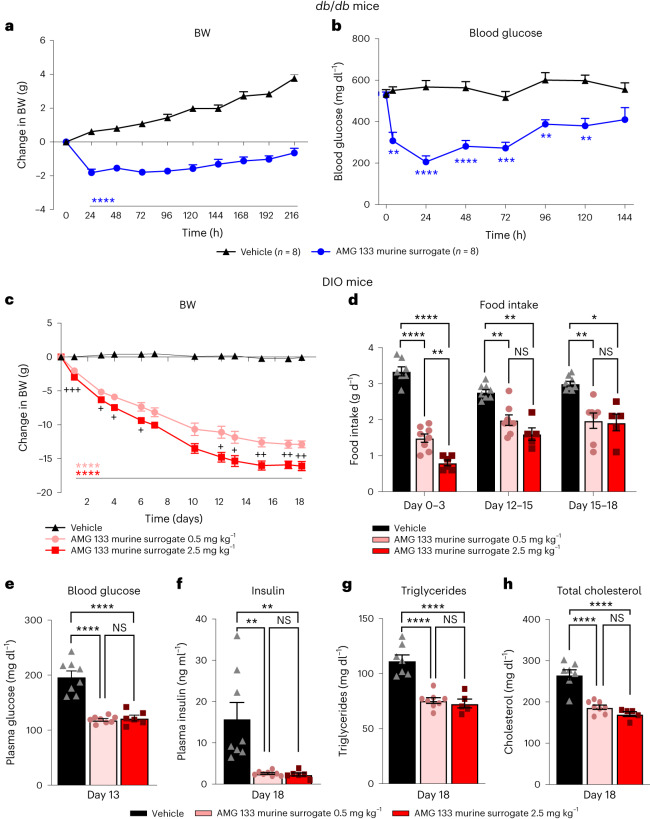
AMG 133 murine surrogate reduced body weight in mouse models of obesity. **a**,**b**, Changes in BW (**a**) and blood glucose (**b**) in male *db*/*db* mice following a single i.p. injection of the AMG 133 murine surrogate; *n* = 8 per group. **c**–**h**, Dose-dependent reductions in BW (**c**) and food intake (**d**) with the AMG 133 murine surrogate in DIO mice. Blood glucose (**e**), fasting insulin (**f**), triglycerides (**g**) and total cholesterol (**h**) decreased in DIO mice treated with the AMG 133 murine surrogate. **P < 0.01, ***P < 0.001 and ****P < 0.0001 versus vehicle (**a**–**c**) or versus vehicle and between doses (**d**–**h**). NS, not significant. ^+^*P* < 0.05, ^++^*P* < 0.01 and ^+++^*P* < 0.001 between doses (**c**). Data represent group mean ± s.e.m. *n* = 5–8 per group depending on group and dataset. Statistical tests were all adjusted for multiple comparisons and included two-way analysis of variance (ANOVA) (**a**–**c**), mixed-effects analysis (**d**) and ordinary one-way ANOVA (**e**–**h**). Source data

The AMG 133 murine surrogate shows a rapid and sustained effect on lowering BW (Fig. 2a) and blood glucose levels (Fig. 2b) in male *db*/*db* mice following a single intraperitoneal (i.p.) injection. In mice treated with 2 mg kg^−1^ AMG 133 murine surrogate, maximal BW loss is achieved within 24 h after the injection and remains significant (*P* < 0.001) up to 216 h after the injection (Fig. 2a); the glucose-lowering effect is observed as early as 4 h after the injection and lasts for 144 h (Fig. 2b). A dose-dependent BW loss is observed with AMG 133 murine surrogate in DIO mice (Fig. 2c). Both the low dose (0.5 mg kg^−1^) and high dose (2.5 mg kg^−1^) of AMG 133 murine surrogate significantly reduces BW from day 1 until day 18. Food intake significantly decreases in a dose-proportional manner, with both dose groups showing substantial reductions during each of the 3-d assessments, with the most pronounced reduction observed on days 0 to 3. (Fig. 2d). Blood glucose, plasma insulin and lipid levels significantly decreased in DIO mice treated with the AMG 133 murine surrogate (Fig. 2e–h).

**Fig. 3:**
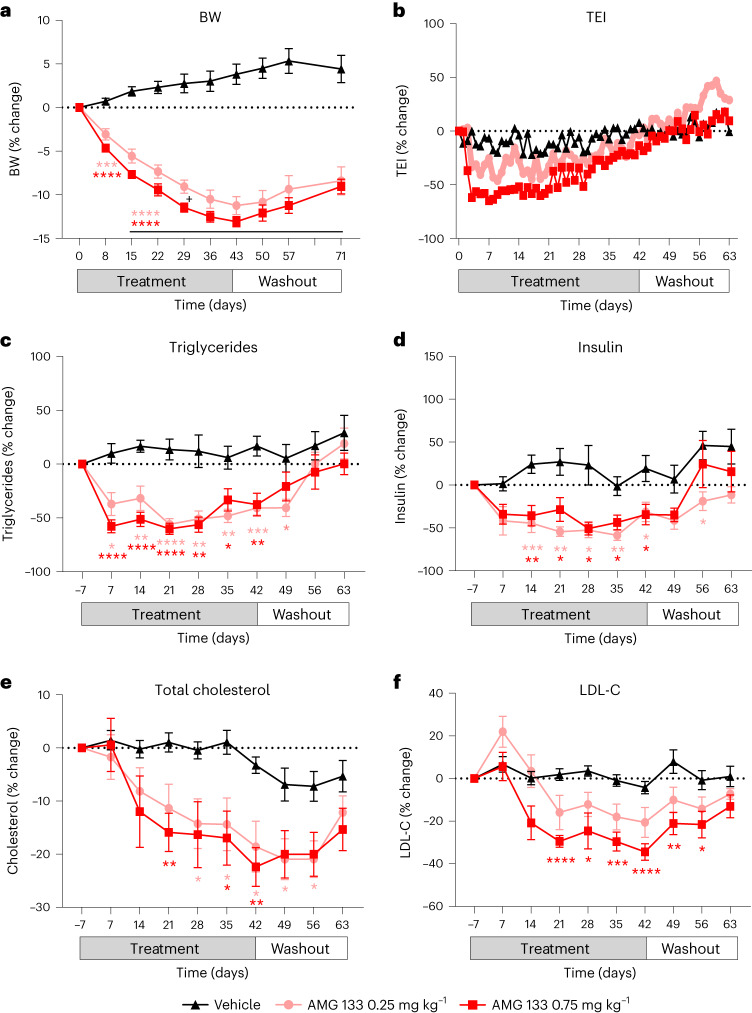
AMG 133 reduced body weight in obese cynomolgus monkeys. **a**–**e**, AMG 133 led to a reduction in BW (**a**), total energy intake (TEI) (**b**), triglycerides (**c**), fasting insulin (**d**), total cholesterol (**e**) and LDL-C (**f**). *P < 0.05, **P < 0.01, ***P < 0.001 and ****P < 0.0001 versus vehicle. ^+^*P* < 0.05 for AMG 133 0.25 mg kg^−1^ versus AMG 133 0.75 mg kg^−1^. Data show group mean ± s.e.m.; vehicle *n* = 10, AMG 133 0.25 mg kg^−1^ *n* = 9 and AMG 133 0.75 mg kg^−1^ *n* = 8. Statistical test used was a two-way ANOVA adjusted for multiple comparisons (**a**,**c**–**f**; statistical analysis not performed on TEI). Source data

AMG 133 treatment also leads to a reduction in BW in obese monkeys (Fig. 3). Weekly s.c. dosing of either 0.25 mg kg^−1^ or 0.75 mg kg^−1^ AMG 133 for 6 weeks leads to a reduction in BW (Fig. 3a), total energy intake (Fig. 3b), fasting triglycerides (Fig. 3c), fasting insulin (Fig. 3d), total cholesterol (Fig. 3e) and low-density lipoprotein cholesterol (LDL-C) (Fig. 3f). BW decreased by 11% and 13% from baseline at the end of the treatment period in the group of animals treated with 0.25 mg kg^−1^ and 0.75 mg kg^−1^ AMG 133, respectively. In the first 3 weeks of dosing, there was a dose-dependent reduction in total energy intake. For fasting insulin and lipids levels, a similar reduction from baseline levels was observed with both 0.25 mg kg^−1^ and 0.75 mg kg^−1^ AMG 133.

### AMG 133 clinical study

**Extended Data Fig. 3:**
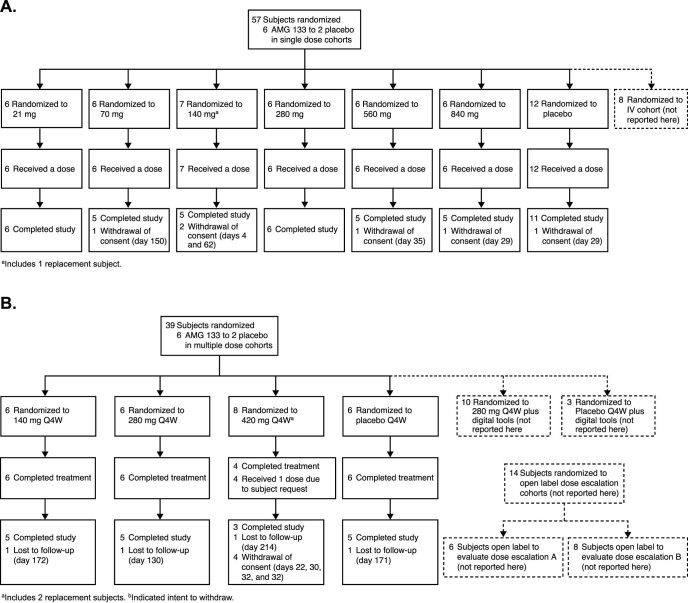
Phase 1 trial patient disposition. (**a**) SAD participant flow chart (CONSORT diagram). (**b**) MAD participant flow chart (CONSORT diagram).

A phase 1, randomized, double-blind, placebo-controlled study was conducted to evaluate the safety, tolerability, PK and PD of single ascending doses (SADs) and multiple ascending doses (MADs) of AMG 133 in adult participants with obesity (NCT04478708). The primary end points of the study were safety and tolerability. PK and immunogenicity were secondary end points and PD biomarkers, including weight were exploratory end points. A total of 49 participants were enroled into seven SAD cohorts, randomized to receive AMG 133 ranging from 21 to 840 mg (*n* = 37) or placebo (*n* = 12) and followed for up to 150 d. Twenty-six participants were enroled into three MAD cohorts, randomized to receive 140, 280 or 420 mg AMG 133, or placebo and followed until day 207 (Extended Data Fig. 3). In the MAD cohorts, AMG 133 or placebo was administered s.c. every 4 weeks on days 1, 29 and 57 for a total of three doses. All participants in the 140 and 280 mg AMG 133 MAD cohorts and the placebo cohort received all three scheduled doses, whereas four out of eight participants in the 420 mg AMG 133 MAD cohort completed all three doses (Extended Data Fig. 3). The baseline demographic characteristics are summarized in Table 1. The mean age ranged from 45.5 to 53.8 years in the SAD cohorts and from 40.3 to 51.6 years in the MAD cohorts. The mean body mass index (BMI) ranged from 32.5 to 34.8 kg m^−2^ in the SAD cohorts and from 32.5 to 34.2 kg m^−2^ in the MAD cohorts. The participants did not have a history of diabetes mellitus and the mean haemoglobin A1c (HbA1c) ranged from 5.4 to 5.6% in the SAD cohorts and from 5.5 to 5.6% in the MAD cohorts.

**Table 1:** Baseline demographics and clinical characteristics of participants receiving single ascending doses (**a**) or multiple ascending doses (**b**) of AMG 133 or placebo

a							
	Placebo (*n* = 12)	21 mg (*n* = 6)	70 mg (*n* = 6)	140 mg (*n* = 7)	280 mg (*n* = 6)	560 mg (*n* = 6)	840 mg (*n* = 6)
Age (years)	45.7 ± 13.4	53.8 ± 9.5	47.8 ± 12.9	48.0 ± 11.9	45.5 ± 11.6	50.2 ± 7.2	45.7 ± 13.7
Males	8 (66.7)	1 (16.7)	5 (83.3)	4 (57.1)	4 (66.7)	5 (83.3)	5 (83.3)
Race							
American Indian or Alaska Native	0 (0.0)	0 (0.0)	0 (0.0)	0 (0.0)	0 (0.0)	1 (16.7)	0 (0.0)
Asian	0 (0.0)	0 (0.0)	0 (0.0)	0 (0.0)	0 (0.0)	0 (0.0)	1 (16.7)
Black (or African American)	4 (33.3)	2 (33.3)	2 (33.3)	5 (71.4)	3 (50.0)	0 (0.0)	0 (0.0)
White	8 (66.7)	4 (66.7)	4 (66.7)	2 (28.6)	3 (50.0)	5 (83.3)	5 (83.3)
Weight (kg)	97.2 ± 12.4	94.9 ± 11.0	104.5 ± 13.0	95.5 ± 14.5	101.4 ± 9.7	107.4 ± 21.0	98.8 ± 13.5
BMI (kg m^−2^)	32.8 ± 2.1	33.8 ± 2.6	32.5 ± 4.0	33.9 ± 1.8	34.8 ± 4.0	34.3 ± 3.6	32.5 ± 2.3
HbA1c (%)	5.38 ± 0.35	5.62 ± 0.22	5.45 ± 0.41	5.61 ± 0.48	5.40 ± 0.74	5.48 ± 0.26	5.57 ± 0.27

b				
	Placebo (*n* = 6)	140 mg (*n* = 6)	280 mg (*n* = 6)	420 mg (*n* = 8)
Age (years)	45.7 ± 14.0	40.3 ± 16.6	44.5 ± 13.8	51.6 ± 12.8
Males	2 (33.3)	5 (83.3)	4 (66.7)	1 (12.5)
Race				
American Indian or Alaska Native	0 (0.0)	0 (0.0)	0 (0.0)	0 (0.0)
Asian	0 (0.0)	1 (16.7)	0 (0.0)	0 (0.0)
Black (or African American)	1 (16.7)	3 (50.0)	1 (16.7)	1 (12.5)
White	5 (83.3)	2 (33.3)	5 (83.3)	7 (87.5)
Weight (kg)	98.9 ± 15.8	101.5 ± 8.3	98.7 ± 16.5	90.5 ± 10.3
BMI (kg m^−2^)	34.2 ± 3.7	34.1 ± 2.9	33.4 ± 3.6	32.5 ± 2.6
Waist circumference (cm)	108.1 ± 16.7	105.2 ± 10.0	106.2 ± 20.9	102.4 ± 10.8
HbA1c (%)	5.50 ± 0.20	5.60 ± 0.48	5.57 ± 0.33	5.58 ± 0.27

Data are mean ± s.d. or number (%) of participants.

For the SAD cohorts (**a**), a 70 mg i.v. cohort (*n* = 8) to determine bioavailability was not included. For the MAD cohorts (**b**), one cohort (280 mg × 3; *n* = 13) to evaluate digital tools was not included. Two open-label cohorts (final dose level did not exceed 420 mg, *n* = 14) to evaluate titration were not included.

### Clinical safety profile

AMG 133 has an acceptable safety and tolerability profile. There were no notable differences between treatment groups for clinical safety laboratory parameters, including electrolytes, kidney function and haematology. In the SAD cohorts, there was one report of amylase and lipase elevation that resolved without clinical sequelae in a participant receiving a single dose of 140 mg (Table 2a). There was one report of amylase and lipase elevation in the 140 mg MAD group that also resolved without clinical sequelae (Table 2b). There were no notable differences in electrocardiogram parameters between groups.

**Table 2:** Gastrointestinal-related treatment-emergent adverse events in humans after a single dose (**a**) or multiple ascending doses (**b**) of placebo and AMG 133

a							
	Placebo (*n* = 12)	21 mg (*n* = 6)	70 mg (*n* = 6)	140 mg (*n* = 7)	280 mg (*n* = 6)	560 mg (*n* = 6)	840 mg (*n* = 6)
Number of individuals reporting TEAEs	3 (25.0)	0 (0.0)	2 (33.3)	5 (71.4)	6 (100.0)	5 (83.3)	6 (100.0)
GI disorders							
Diarrhoea	2 (16.7)	0 (0.0)	0 (0.0)	0 (0.0)	0 (0.0)	0 (0.0)	0 (0.0)
Dyspepsia	1 (8.3)	0 (0.0)	1 (16.7)	4 (57.1)	0 (0.0)	0 (0.0)	0 (0.0)
Abdominal discomfort	0 (0.0)	0 (0.0)	0 (0.0)	2 (28.6)	0 (0.0)	0 (0.0)	0 (0.0)
Constipation	1 (8.3)	0 (0.0)	0 (0.0)	0 (0.0)	1 (16.7)	0 (0.0)	3 (50.0)
Gastroesophageal reflux disease	0 (0.0)	0 (0.0)	0 (0.0)	0 (0.0)	1 (16.7)	0 (0.0)	0 (0.0)
Nausea	3 (25.0)	0 (0.0)	2 (33.3)	4 (57.1)	4 (66.7)	5 (83.3)	5 (83.3)
Vomiting	0 (0.0)	0 (0.0)	1 (16.7)	4 (57.1)	5 (83.3)	5 (83.3)	4 (66.7)
GI safety laboratory							
Amylase elevation	0 (0.0)	0 (0.0)	0 (0.0)	1 (14.3)	0 (0.0)	0 (0.0)	0 (0.0)
Lipase elevation	0 (0.0)	0 (0.0)	0 (0.0)	1 (14.3)	0 (0.0)	0 (0.0)	0 (0.0)
Hepatic enzyme elevation	0 (0.0)	0 (0.0)	0 (0.0)	0 (0.0)	1 (16.7)	0 (0.0)	0 (0.0)

b				
	Placebo (*n* = 6)	140 mg (*n* = 6)	280 mg (*n* = 6)	420 mg (*n* = 8)
Number of individuals reporting TEAEs	3 (50.0)	6 (100.0)	6 (100.0)	8 (100.0)
GI disorders				
Diarrhoea	0 (0.0)	1 (16.7)	0 (0.0)	2 (25.0)
Dyspepsia	0 (0.0)	1 (16.7)	0 (0.0)	1 (12.5)
Abdominal distension	0 (0.0)	1 (16.7)	0 (0.0)	1 (12.5)
Abdominal pain upper	0 (0.0)	0 (0.0)	0 (0.0)	1 (12.5)
Constipation	0 (0.0)	2 (33.3)	1 (16.7)	0 (0.0)
Nausea	1 (16.7)	5 (83.3)	4 (66.7)	8 (100.0)
Vomiting	0 (0.0)	4 (66.7)	5 (83.3)	6 (75.0)
GI safety laboratory				
Amylase elevation	0 (0.0)	1 (16.7)	0 (0.0)	0 (0.0)
Lipase elevation	0 (0.0)	1 (16.7)	0 (0.0)	0 (0.0)

Data show number (%) of participants with the event of interest.

The most common treatment-emergent adverse events (TEAEs) were related to GI symptoms, specifically nausea and vomiting (Table 2b), which were generally mild and resolved within 48 h after administration of AMG 133. No severe or serious TEAEs or TEAEs leading to permanent discontinuation were reported during the study (Extended Data Table 1). In the SAD cohorts, TEAEs were mild, except in one participant in the 140 mg group who reported moderate TEAEs due to gastrointestinal (GI) symptoms and one participant in the 280 mg group who had moderate symptoms related to a COVID infection, including elevated liver enzymes (Table 2a). In the MAD cohorts, all TEAEs were reported as mild. In the MAD cohorts, all six participants completed treatment in the placebo, 140 mg and 280 mg groups, and four out of eight participants completed treatment in the 420 mg group. The other four participants in the 420 mg cohort received one dose of AMG 133 (Extended Data Fig. 3). Of the participants who received at least two doses, 68% of them had GI-related TEAEs after the first dose and only 9% of participants had GI-related TEAEs after subsequent doses. Even though a reduction of fasting glucose levels were observed with AMG 133 (see ‘Clinical PK and PD’), there were no reported adverse events related to hypoglycemia.

No clinically meaningful changes in blood pressure were observed after single or multiple doses of AMG 133 (Extended Data Table 2). Heart rate increased within the normal range after the single dose of AMG 133 in the SAD cohorts. These changes were not dose-dependent; for example, at day 5, the heart rate increase was 13.6 ± 8.2 beats per minute (b.p.m.) in the 21 mg cohort and 4.2 ± 8.7 b.p.m. in the 840 mg cohort. Similarly, in the MAD cohorts, the heart rate increased in cohorts receiving AMG 133, but these changes were also within the normal range and were not dose dependent. At day 7, the heart rate (s.d.) increased in the 140, 280 and 420 mg group to 85.2 (6.4), 78.5 (6.1) and 83.5 (12.3) b.p.m., respectively (data not included in the table). An additional increase in heart rate was not observed after the second or third dose of AMG 133. Neither tachycardia nor palpitations were observed as adverse events in the SAD or MAD cohorts.

### Clinical PK and PD

**Fig. 4:**
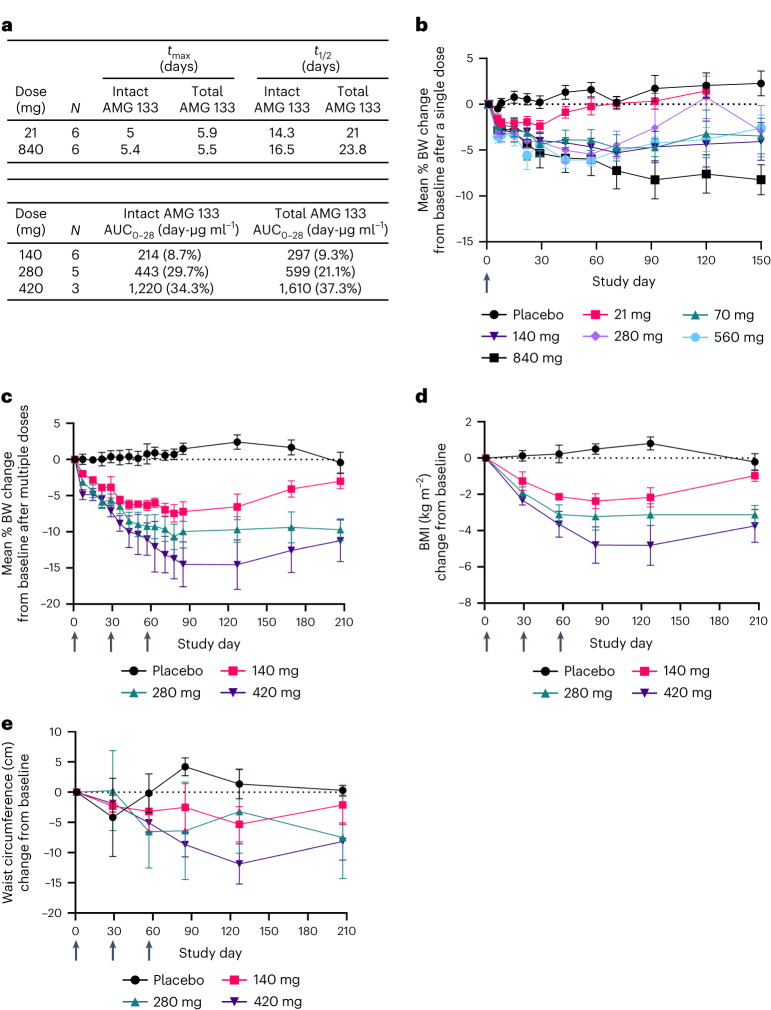
Clinical PK and PD profiles of AMG 133 in humans. **a**, AMG 133 PK parameters; *t*_max_ is presented as the median and *t*_1/2_ is presented as the geometric mean. AUC_0–28_ is presented as the geometric mean (CV%) after the last dose of AMG 133 on day 57 for MAD cohorts. **b**–**e**, Mean (s.e.m.) percent change from baseline in BW after single doses, *n* = 6–7 for AMG 133 and *n* = 12 for placebo at day 1 (**b**) and multiple doses, *n* = 6–8 for AMG 133 and *n* = 6 for placebo at day 1 (**c**). Mean (s.e.m.) change from baseline in BMI, *n* = 6–8 for AMG 133 and *n* = 6 for placebo at day 1 (**d**) and waist circumference, *n* = 6–8 for AMG 133 and *n* = 6 for placebo at day 1 (**e**) after multiple doses of AMG 133. Arrows indicate when the investigational product was administered: at day 1 in the SAD cohorts (**b**) and at day 1, 29 and 57 in the MAD cohorts (**c**–**e**). Source data

Across the doses evaluated in the SAD cohorts, AMG 133 reached maximum concentrations (median *t*_max_) at approximately 4 to 7 d post-dose. The mean *t*_1/2_ ranged from approximately 14 to 16 d for intact AMG 133 and 21 to 24 d for total AMG 133. AMG 133 plasma exposures seemed to increase approximately dose-proportionally for both intact and total AMG 133 (Fig. 4a). In the MAD cohorts, the median *t*_max_ ranged from 4 to 6 d post-dose, consistent with the SAD cohorts. The intact and total AMG 133 exposures (AUC_0–28d_) following the last dose on day 57 are presented in Fig. 4a.

Treatment with AMG 133 results in dose-dependent decreases in mean BW, BMI and waist circumference from baseline (Fig. 4b–e). At the lowest single dose of 21 mg, the mean percent change of BW from baseline ranged from −1.6% at day 6 to −2.4% at day 29. The highest single dose of 840 mg resulted in a −2.8% change at day 6 and −8.2% at day 92, suggesting a prolonged weight-reducing effect after a single dose of AMG 133 compared to a mean percent change in BW of −0.5% at day 6, 0.2% at day 29 and 1.7% at day 92 in the placebo group. The decreases in BW were maintained until day 120 for doses tested above 21 mg (Fig. 4b).

In the MAD cohorts, the 140 mg dose Q4W resulted in a mean percent BW change of −2.0% at day 7 and −7.4% after three doses at day 78. The highest MAD dose of 420 mg Q4W resulted in a BW change of −4.9% at day 7 and −14.5% by day 85. In comparison, the placebo Q4W group had a mean percent BW change of 0.04% at day 7 and 1.5% at day 85. The reduction in BW after three Q4W doses of AMG 133 was maintained through 70 d following the third dose in each group and in the 420 mg Q4W group, the BW remained reduced up to −11.2%, 150 d after the last AMG 133 dose (Fig. 4c). The change from baseline in BMI followed a similar pattern as the change in BW (Fig. 4d). A dose-dependent decrease in waist circumference was observed with MAD 140 mg and 420 mg, whereas fluctuations in waist circumference changes were displayed with MAD 280 mg (Fig. 4e), which may reflect the variability of these measurements in a small sample size. Statistical testing for changes in BW from baseline to day 29 in the SAD cohorts and for changes in BW, BMI and waist circumference from baseline to day 85 in the MAD cohorts is provided in Extended Data Table 3a.

**Fig. 5:**
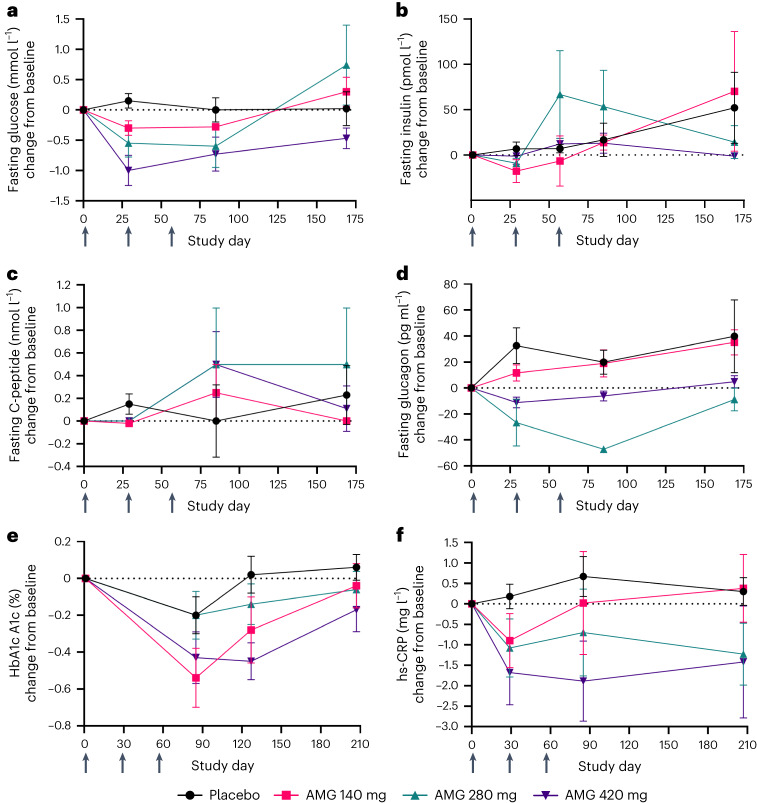
Changes in metabolic and inflammatory parameters in humans with multiple ascending doses of AMG 133. **a**–**f**, Mean (s.e.m.) change from baseline in fasting glucose, *n* = 5–8 for AMG 133 and *n* = 6 for placebo at day 1 (**a**), fasting insulin, *n* = 4–8 for AMG 133 and *n* = 5 for placebo at day 1 (**b**), fasting C-peptide *n* = 3–8 for AMG 133 and *n* = 5 for placebo at day 1 (**c**), fasting glucagon, *n* = 3–8 for AMG 133 and *n* = 5 for placebo at day 1 (**d**), HbA1c, *n* = 6–8 for AMG 133 and *n* = 6 for placebo at day 1 (**e**) and hs-CRP, *n* = 6–8 for AMG 133 and *n* = 6 for placebo at day 1 (**f**) after MADs of AMG 133. Arrows indicate when the investigational product was administered at day 1, 29 and 57. Source data

Changes in PD biomarkers in the MAD cohorts are shown in Fig. 5. The fasting glucose decreased from baseline in a dose-responsive manner at days 29 and 85, whereas the glucose levels in the placebo group remained close to baseline throughout the same period. Therefore, the effect on fasting glucose seems to be treatment-specific, as the decrease was dose-dependent and glucose levels returned toward or above baseline during the safety follow-up period (day 169; Fig. 5a). The decrease in fasting insulin levels seen in DIO mice and obese monkeys was partially recapitulated in the clinical trial. An early decrease from baseline in fasting insulin was observed at day 29 (Fig. 5b) but was not maintained at day 85 or day 169. No remarkable trends in fasting C-peptide levels were observed (Fig. 5c). An increase from baseline in the fasting glucagon levels in the placebo and 140 mg groups was observed at days 29, 85 and 169. In contrast, there was a reduction in fasting glucagon levels observed with the 280 mg and 420 mg doses normalizing in the safety follow-up period (day 169), suggesting an AMG 133-specific effect (Fig. 5d). HbA1c levels, which were in the normal (non-diabetic) range at baseline, decreased in all three dose groups in response to AMG 133 by day 85, but trended toward baseline during the safety follow-up period at day 207 (Fig. 5e). High-sensitivity C-reactive protein (hs-CRP) was measured as a marker of inflammation. AMG 133 treatment was associated with dose-dependent decreases in hs-CRP levels by day 29 in the MAD cohorts (Fig. 5f). The decrease in hs-CRP levels trended in line with the BW changes for each group (hs-CRP levels were sustained until day 207 at the 280 and 420 mg doses but normalized to baseline at the 140 mg dose). Statistical testing for PD biomarkers in the MAD cohorts is provided in Extended Data Table 3b.

**Extended Data Fig. 4:**
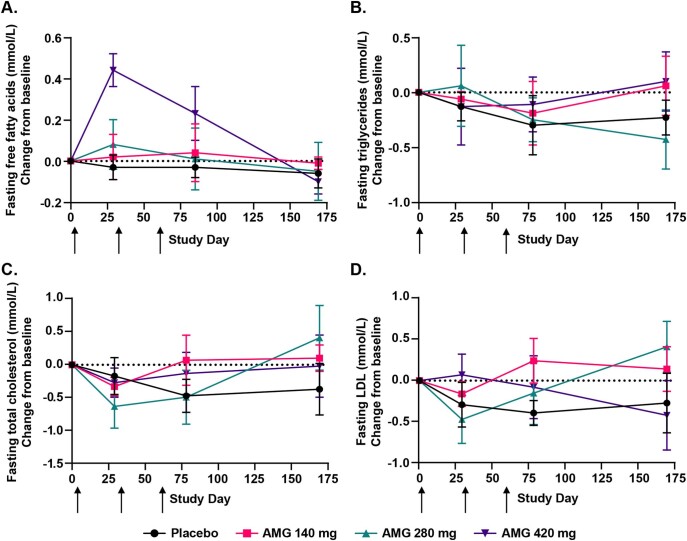
Changes in free fatty acids and lipid parameters in humans with multiple ascending doses of AMG 133. (**a–d**) Mean (SE) change from baseline in fasting free fatty acids, n = 6–8 for AMG 133 and n = 6 for placebo at Day 1 (A), fasting triglycerides, n = 6 for AMG 133 and n = 6 for placebo at Day 1 (B), fasting total cholesterol, n = 6–8 for AMG 133 and n = 6 for placebo at Day 1 (C), fasting LDL, n = 6 for AMG 133 and n = 6 for placebo at Day 1 (D) after multiple ascending doses of AMG 133 or placebo. Arrows indicate when the investigational product was administrated: at day 1, 29, and 57. Source data

An increase in free fatty acids was observed in the AMG 133 treatment groups compared to the placebo group. The 420 mg group showed a greater increase compared to the 140 mg and 280 mg groups at days 29 and 78, but levels returned to or below baseline by day 169 (Extended Data Fig. 4a). Lipid parameters were also monitored throughout the study (Extended Data Fig. 4b–d). Transient decreases in total cholesterol, LDL-C and triglycerides were observed anywhere between day 29 and 78 in all treatment groups, including the placebo group. Statistical testing results are provided in Extended Data Table 3c.

## Discussion

Here, we report that the bispecific molecule AMG 133, which has dual GIPR antagonist and GLP-1R agonist effects in cell-based systems, effectively reduces BW and improves metabolic markers in two preclinical models. In a phase 1 clinical study, AMG 133 had an acceptable safety and tolerability profile along with dose-dependent weight loss. The weight loss in the MAD cohorts was maintained up to 150 d after the last dose.

**Extended Data Fig. 5:**
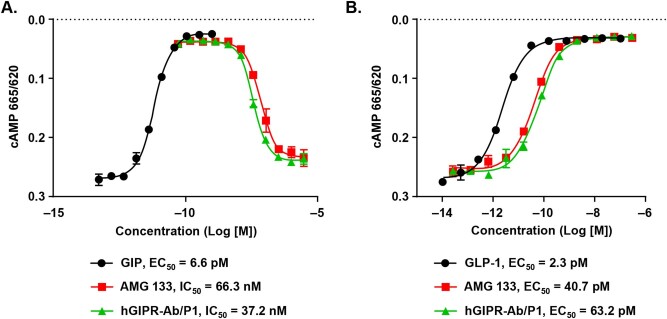
In vitro functional activity comparison between AMG 133 and hGIPR-Ab/P1. (**a**) Representative dose-response curves of cAMP assays with GIP (agonist mode) and AMG 133 or hGIPR-Ab/P1 + 50 pM GIP (antagonist mode) in cells expressing human GIPR. Data are represented as mean ± SEM of n = 2 replicates per treatment. (**b**) Representative dose-response curves of cAMP assays with GLP-1, AMG 133 or hGIPR-Ab/P1 in cells expressing human GLP-1R. Data are represented as mean ± SEM of n = 2 replicates per treatment. Source data

The combination of an anti-GIPR monoclonal antibody (mAb) and a GLP-1RA has been shown to mediate more pronounced weight loss than either agent alone in preclinical obesity models^33^. Furthermore, bispecific molecules with GIPR-Ab conjugated to GLP-1 peptide with functional activities comparable to those of AMG 133 (Extended Data Fig. 5) showed synergistic effects on weight loss and improved multiple metabolic parameters in DIO mice^34^. A mechanistic study suggested that GIPR-Ab/GLP-1 bispecific molecules bind to GIPR and GLP-1R simultaneously and trigger receptor internalization, amplifying endosomal cAMP signalling in cells expressing both receptors^34^. Taken together, these data support the idea that a GIPR-Ab/GLP-1 bispecific molecule would generate greater weight loss compared to either agent administered alone. In the present study, the synergistic effects of GIPR antagonism combined with GLP-1R agonism were replicated with AMG 133 not only in obese mouse models and cynomolgus monkeys, but also confirmed in humans.

In preclinical species and humans, mAbs are known to have minimal brain penetration, at around 0.1–0.4% of mAb plasma concentration^35,36^. The observed reductions in food intake in obese mice (Fig. 2d) and monkeys (Fig. 3b) may be due to the peripheral effects of AMG 133 murine surrogate and AMG 133, respectively. Because of their molecular weight, these compounds likely do not cross the blood–brain barrier. Both GLP-1R and GIPR are expressed outside the blood–brain barrier, including in the area postrema, which is important for food intake^37^.

Strong genetic evidence such as GWAS has identified single-nucleotide polymorphisms in Japanese, European and North American populations showing that lower expression or function of GIPR is associated with lower BMI^27,29,38^ and genetic preclinical models^30,31,39,40^ support the role of GIPR antagonism as a method to promote weight loss and prevent weight gain. It is unclear how to reconcile this genetic evidence with data showing that unimolecular dual GIPR/GLP-1 agonism is associated with increased weight loss over GLP-1R agonism alone^7^. The precise mechanism by which both GIPR antagonism and GIPR agonism result in weight loss in humans, when combined with GLP-1R agonism, is not known; however, previous data suggest that following multiple GIP treatments, GIPR expression is reduced and GIP-stimulated cAMP production is lowered, representing an effective desensitization of GIPR to its ligand^41^.

The GIPR antagonism/GLP-1R agonism approach was validated in animal models, supporting advancement to clinical testing. The findings in humans were mostly consistent with those in cynomolgus monkeys. In participants with obesity, higher doses resulted in higher BW loss after a single dose. In the MAD setting, participants receiving AMG 133 demonstrated sustained weight loss after 12 weeks of treatment. AMG 133 treatment was also associated with changes in glucose homeostasis markers as well as a dose-dependent reduction in levels of the inflammation marker hs-CRP. Even though there was a reduction in cholesterol, LDL and triglycerides level associated with AMG 133 treatment in cynomolgus monkeys, the AMG 133 effects on lowering lipid parameters were inconclusive in the first-in-human (FIH) study due to the large variability associated with the small sample size of each cohort coupled with the observation that the placebo group also had reductions in cholesterol, LDL-C and triglycerides. For the clinical study, participants were excluded if they had high triglycerides (≥5.65 mmol l^−1^ (500 mg dl^−1^)) at screening, which may have limited the ability to detect meaningful reductions in response to AMG 133. PK data from the study demonstrated that maximum plasma concentrations were reached by day 4 to 6 post-dose, with a mean *t*_1/2_ ranging from 14 to 24 d for intact and total AMG 133. The longer half-life of AMG 133 (mean *t*_1/2_ ~ 21 d) along with the duration of weight loss seen after a single dose as well as repeat dosing of AMG 133 enabled less-frequent dosing at every 4 weeks in the MAD study compared to other GLP-1 agonists and dual GIPR/GLP-1 agonists, which require either daily or weekly dosing based on the shorter half-lives of these agents^20,42,43^.

The safety profile of AMG 133 was similar to that of GLP-1RAs, including unimolecular GLP-1/GIPR agonist peptides^6,7^. The most frequently reported adverse events were GI-related, including nausea and vomiting and were mostly mild in severity. No increase in the severity of nausea, vomiting or other TEAEs was observed with increasing doses of AMG 133. GI-related adverse events mainly occurred after the first dose in the MAD cohorts, started 8–12 h post-dose and lasted for about 72 h. While GI-related adverse events are expected with incretin therapeutics, the exact mechanism is unknown. The early onset and resolution of the GI adverse events occurred before the *t*_max_, which was between 4–6 d, suggesting that the GI-related events may not be related to drug exposure. At the highest MAD dose of 420 mg, four participants withdrew from the study before receiving the second dose after reporting mild GI-related adverse events; therefore, intra-patient dose-escalation-based regimens may offer a future advantage in decreasing the first-dose effect seen with AMG 133.

The main limitation of this FIH study was the small sample size. Cautious interpretation of PD effects, including metabolic parameters is thus required. Furthermore, the participants had neither diabetes nor high triglyceride and cholesterol levels, which further limits the generalizability of the metabolic parameter findings.

The clinical study was designed to characterize the safety and tolerability of AMG 133 and hence no active comparator arm was included in the study. Although AMG 133 is a single molecule with multiple targets and the weight loss results from the dual action of GIPR antagonism and GLP-1 agonism, as established in preclinical assessment, the present human trial design did not allow for distinction of the individual contributions of GIPR antagonistic activity and GLP-1 agonist activity to weight loss. The small sample size limits conclusions regarding the generalizability of both PD and safety data.

The overall changes observed in weight and metabolic parameters for both preclinical and clinical studies suggest strong incretin-related effects. Further investigation is still needed to optimize the dosing regimen of AMG 133 for weight loss and other metabolic effects. In conclusion, the safety and tolerability profiles, the longer half-life of AMG 133 allowing for extended dosing intervals and the magnitude and durability of weight loss support continued evaluation of AMG 133 in a phase 2 setting (NCT05669599).

## Methods

### Ethical regulations, randomization and compensation for studies in humans

The research described in this manuscript complies with all relevant ethical regulations. The clinical study was conducted in full accordance with the Declaration of Helsinki and the International Conference on Harmonization Good Clinical Practices Guideline. The study protocol, all amendments and the informed consent form were reviewed and approved by the Institutional Review Boards at each site, including Orange County Research Center, Anaheim Clinical Trials and Clinical Pharmacology of Miami. Written informed consent was obtained from eligible participants before any study-related procedures. After obtaining informed consent, the clinical site registered the participants in the Interactive Response Technology (IRT) by Almac and screened the participants to assess eligibility for participation in the trial. As soon as a participant met the eligibility criteria and was enroled into the trial, the randomization to the treatment group was performed by IRT. The participants were assigned a randomization number based in the sequential order in which they qualified to be randomized. Patient compensation was at the discretion of the clinical sites and is confidential and proprietary information held by the clinical site. Amgen does not decide compensation and is not informed on how the sites decide this.

### Ethical regulations for studies in animals

No statistical methods were used to predetermine sample sizes for preclinical experiments, but our sample sizes are similar to those reported in previous publications^34^.

The PK study in healthy monkeys was performed at MPI Research in accordance with protocols approved by the MPI Research Institutional Animal Care and Use Committee (IACUC). Animal care was conducted in accordance with the Guide for the Care and Use of Laboratory Animals^44^. The study in obese cynomolgus monkeys was performed at Kunming Biomed International (KBI) according to research protocols approved by the KBI IACUC. Animals were housed in an Association for Assessment and Accreditation of Laboratory Animal Care (AAALAC) International-accredited facility and cared for in accordance with guidance^44^. Data collection and analysis in obese cynomolgus monkeys were performed blind to the conditions of the experiments.

The *db*/*db* and DIO mouse studies as well as PK study in healthy mice were performed at Amgen, an AAALAC International-accredited facility. All study procedures were approved by the Amgen IACUC and conducted in accordance with guidance^44^. The lighting in animal-holding rooms was maintained on a 12-h light–dark cycle and the ambient temperature and humidity range was 68 to 79 °F and 30 to 70%, respectively. Data collection and analysis were not performed blind to the conditions of the experiments.

### AMG 133 bispecific molecule synthesis

AMG 133 synthesis conditions were similar to those used previously for other GIPR-Ab/GLP-1 bispecific molecule synthesis^34^. An anti-GIPR-Ab with a specific cysteine mutation at the E384 position was incubated with a solution of 2.5 mM cystamine and 2.5 mM cysteamine in 40 mM HEPES buffer, pH 8.2 at 2.5 mg ml^−1^ concentration for 15–20 h (Extended Data Fig. 1). The reaction mixture was passed through a sterile filter and diluted with 100 mM sodium acetate (NaOAc) buffer, pH 5.0. Purification of the reaction mixture was performed by cation exchange chromatography (GE 240 ml SP/HP; A, 20 mM NaOAc, pH 5.0; B, A + 1.0 M NaCl, 0–25% over 10 CV, 20 ml min^−1^). The main peak that contained bis-cysteamine-capped GIPR-Ab cys-mAb was gathered and buffer exchanged into 10 mM sodium acetate with 9% sucrose, pH 5.2, using tangential flow filtration (Millipore Pellicon 3, Ultracel 30 kDa Membrane, 0.11 m²).

**Extended Data Fig. 6:**
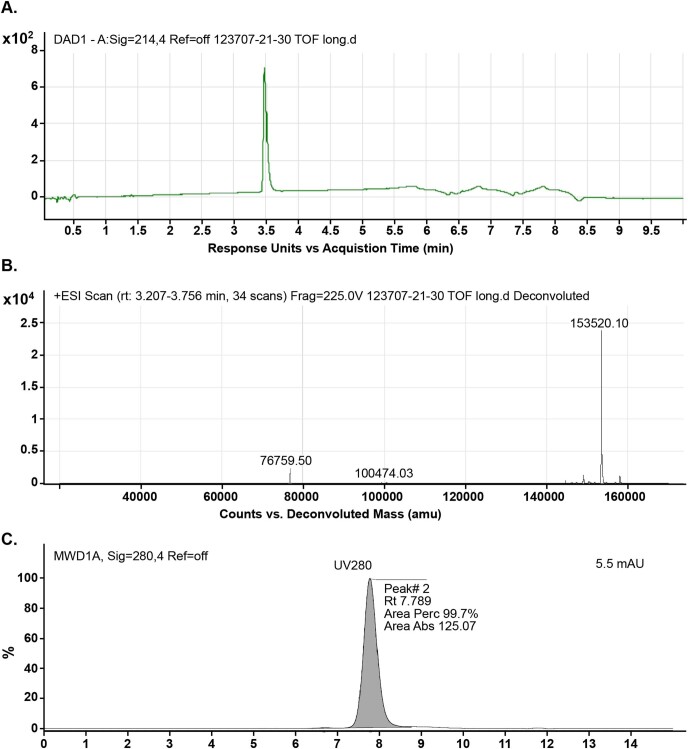
The HPLC-TOF-MS spectrum and SEC chromatograph of AMG 133. (**a**) HPLC-TOF chromatogram, UV 214 nm. (**b**)TOF-MS, full spectrum. (**C**) SEC chromatogram, UV 280 nm.

The resulting solution of GIPR-Ab cys-mAb (6 mg ml^−1^ in 10 mM sodium acetate with 9% sucrose) was partially reduced using tris (2-carboxyethyl) phosphine (4 eq.) at room temperature (RT) for 60–90 min. The protein was buffer exchanged into clean 10 mM NaOAc, 9% sucrose, pH 5.2, then concentrated to 10 mg ml^−1^. The reduced GIPR-Ab cys-mAb was then diluted with 50 mM sodium phosphate buffer containing 2 mM EDTA pH 7.5 to 3.0 mg ml^−1^ and treated with dehydroascorbic acid (BioSynth International (8 eq.)) at RT for 90 min. Bromoacetyl-GLP-1 analogue peptide (4 eq.) was added and the solution was incubated at RT for 15–20 h. The solution was filtered (cellulose acetate, 0.22 µM) and diluted with 20 mM Tris-HCl, pH 7.0. The reaction mixture was purified by cation exchange chromatography (GE 240 ml SP HP column; A, 20 mM Tris-HCl, pH 7.0; B, A + 0.5 M NaCl, 0–10% B over 120 min, 20 ml min^−1^), then buffer exchanged into 10 mM NaOAc, 9% sucrose, pH 5.2 using tangential flow filtration. After concentrating to 25 mg ml^−1^, the conjugated material was characterized by HPLC-TOF–MS (Agilent PLRP-S 4,000 Å, 5.0 µM, 2.1 × 50 mm column; A, 0.1% formic acid/H_2_O; B, 0.1% formic acid/CH_3_CN, 0.8 ml min^−1^, 10–50% B over 5 min) and size exclusion chromatography (SEC; Tosoh QC-PAK GFC 300 7.8 mm × 15 cm column; mobile phase, 0.17 M potassium phosphate, 0.21 M KCl, 15% (*v*/*v*) iPrOH, pH 7.0 over 15 min at 0.5 ml min^−1^). The conjugate molecular weight was confirmed by HPLC-TOF–MS (calculated molecular weight, 153,514; found molecular weight, 153,520). The purity of the conjugate by SEC was at 99.7% monomer. The SEC chromatograph and HPLC-TOF–MS spectrum are summarized in Extended Data Fig. 6.

Reverse-phase HPLC-TOF was run on an Agilent 1290 series UPLC connected to an Agilent 6224 TOF–MS system using an Agilent PLRP-S 4,000 Å, 5.0 μm, 2.1 × 50 mm column. The mobile phases consisted of (A) water and (B) acetonitrile, both with 0.1% formic acid as a modifier, running a gradient of 10–50% B over 5 min, followed by two column flushes at 20–60% B over 1 min each before re-equilibration at 10% B for 1 min. Settings for MS-TOF included Vcap, 5,000 V; fragmentor, 225 V; skimmer, 100 V; OCT RF Vpp, 675 V; and acquisition range, 800–6,000 *m*/*z*. The SEC was run on an Agilent 1260 Bio-Inert HPLC equipped with a Tosoh QC-PAK GFC 300 7.8 mm × 15 cm column, running an isocratic mobile phase consisting of 0.17 M potassium phosphate, 0.21 M KCl and 15% (*v*/*v*) iPrOH, pH 7.0 over 15 min at 0.5 ml min^−1^.

### Cell lines

Parental CHO-K1 and HEK 293T cells were obtained from ATCC (CCL-61 and CRL-3216). CHO cells stably expressing human, mouse, rat and monkey GLP-1R, HEK 293T cells stably expressing human or monkey GIPR and CHO-AM1D cells stably expressing mouse or rat GIPR were all generated at Amgen^34^. CHO stably expressing human GLP-1R cells or mouse GLP-1R cells were cultured in Ham’s F12 medium supplemented with 1% penicillin/streptomycin/l-glutamine (PSG), 10% fetal bovine serum (FBS) and 250 mg ml^−1^ zeocin (Thermo Fisher). CHO-AM1D cells expressing rat GLP-1R were maintained in Dulbecco’s modified Eagle’s medium (DMEM), with 1% PSG, 10% dialysed FBS, 1% nonessential amino acids (NEAAs) and 1 mM sodium pyruvate (Thermo Fisher). CHO-AM1D cells stably expressing monkey GLP-1R cells were cultured in DMEM supplemented with 1% PSG, 10% dialysed FBS, 1% NEAAs, 1 mM sodium pyruvate, 1% sodium hypoxanthine and thymidine supplement (HT supplement) and 400 mg ml^−1^ hygromycin (Thermo Fisher). HEK 293T cells overexpressing human GIPR were cultured in DMEM, 1% PSG, 10% FBS and 5 mg ml^−1^ puromycin (Thermo Fisher). CHO-AMID mouse and rat GIPR cells were cultured in DMEM supplemented with 1% PSG, 10% dialysed FBS, 1% NEAAs, 1 mM sodium pyruvate, 1% HT supplement and 400 mg ml^−1^ hygromycin (Thermo Fisher). HEK 293T monkey GIPR cells were cultured in DMEM supplemented with 1% PSG, 10% FBS and 2 mg ml^−1^ puromycin (Thermo Fisher). All cells were cultured in humidified incubators maintained at 37 °C and 5% CO_2_.

### In vitro cAMP assay

The in vitro cAMP assay was conducted as previously reported^34^. Data collection and analysis were not performed blind to the conditions of the experiments. To assess the GLP-1R agonist activity of AMG 133 or the murine surrogate, CHO cells stably expressing human, mouse, rat or monkey GLP-1R were used in a homogeneous time-resolved fluorescence assay (Cisbio)^34^. Serial diluted AMG 133 or murine surrogate were incubated with 40,000 cells in F12 medium containing 0.1% bovine serum albumin (BSA), 500 mM 3-isobutyl-1-methylxanthine for 15 min at 37 °C (Sigma). Cells were then lysed with lysis buffer containing cAMP-d2 and cAMP cryptate (Cisbio) and incubated for 1 h at RT before measurement in the EnVision plate reader (PerkinElmer). The cAMP levels are expressed as a fluorescence ratio of 665/620 nm. To determine the GIPR antagonist activity, HEK 293T cells stably expressing human or monkey GIPR and CHO-AM1D stably expressing mouse or rat GIPR were used to measure cAMP activity. Serial diluted AMG 133 or murine surrogate were incubated with 30,000 cells in F12 medium containing 0.1% BSA and 500 mM 3-isobutyl-1-methylxanthine for 30 min at 37 °C before treatment with GIP at 50 pM in the HEK 293T cells and 90 pM in the CHO-AM1D cells for an additional 30 min at 37 °C. Cells were then lysed in lysis buffer containing cAMP-d2 and cAMP cryptate (Cisbio) for 1 h at RT. The fluorescence was measured in an EnVision plate reader (PerkinElmer) and the cAMP levels were expressed as a ratio of 665/620 nm. All cAMP data were analysed using GraphPad Prism v.8.4.3 software, log (agonist or inhibitor) versus response, variable slope (four parameters).

### Bioanalytical methods and PK

The concentrations of AMG 133 in mouse and cynomolgus monkey plasma were determined by ELISA methods developed to monitor intact AMG 133 (GIPR-Ab with attached GLP-1 analogue peptides) and total AMG 133 (GIPR-Ab with or without GLP-1 analogue peptides). The analytical range for both assays was from 30 to 2,000 ng ml^−1^. The ELISA reagents and assay for quantitation of intact AMG 133 were consistent with previously published methods^34^, with substitution of AMG 133 for GIPR-Ab/GLP-1 bispecific molecules used in the earlier work. Briefly, AMG 133 in plasma samples was captured on microtiter plates coated with a mouse mAb against human IgG Fc (Clone No. 1.35, Amgen) at 2 µg ml^−1^. A biotinylated mouse mAb against GLP-1 (Clone No. 4, cat. no. ABS 033-04-02, Thermo Fisher Scientific) at 200 ng ml^−1^ and a streptavidin-horseradish peroxidase conjugate (R&D Systems) at 1:1,000 dilution were sequentially added for detection of bound AMG 133. A chromogenic reaction was produced by addition of a tetramethylbenzidine enzyme substrate (SeraCare) and absorbance at 450 nm was measured using a plate reader (Molecular Devices). AMG 133 concentrations were determined by interpolation from a calibration curve fitted to a four-parameter regression model using Watson LIMS (v.7.4; Thermo Fisher).

The total AMG 133 ELISA followed a similar procedure as the intact assay except for the detection reagent. Detection of AMG 133 in the total assay was achieved with an HRP-conjugated mouse mAb against human IgG Fc (Clone No. 21.1, Amgen) at 30 ng ml^−1^. PK parameters were estimated from individual plasma concentration–nominal time data using standard noncompartmental analysis in Phoenix WinNonlin v.6.4 (Certara).

### Non-obese mice

Animals used in the study were male CD-1 mice at 8–12 weeks of age and weighing approximately 30 g (Charles River Laboratories). Following a 1-week acclimation, mice (*n* = 3) received a single i.v. bolus dose of AMG 133 at 5 mg kg^−1^ via the lateral tail vein. Blood samples were collected by submandibular venipuncture at predetermined time points up to 14 d after the dose. Whole blood was placed into Microvette 500 μl K3 EDTA plasma separator tubes (20.1341.102, Sarstedt), gently mixed by 8–10 manual inversions and centrifuged at 11,500*g* at 4 °C for 5 min. The resulting plasma was stored at −70 °C (±10 °C) until analysis.

### Non-obese cynomolgus monkeys

Details of the PK experiments were similar to those previously published^34^. Young adult females weighing 2–3 kg were used from the MPI Research stock colony of naive cynomolgus monkeys (*Macaca* *fascicularis*). Monkeys were housed individually in stainless-steel cages. Lighting was provided for approximately 12 h per day via an automatic timer and environmental enrichment was provided. Water was available ad libitum. Food was offered twice daily (Lab Diet Certified Primate Diet 5048, PMI Nutrition International). Temperature and humidity were controlled between 64–79 °F and 30–70%, respectively. There was an 8-h fasting period before dosing, after which female monkeys (*n* = 3) received a single s.c. bolus dose of AMG 133 at 3 mg kg^−1^ in the scapular region on the back. Blood samples (~1 ml) were collected from the femoral vein/artery at predetermined time points up to 35 d after the dose. Blood samples were processed to K2 EDTA plasma and stored at −70 °C (±10 °C) until analysis.

### *db*/*db* mice

The *db*/*db* mouse study was conducted at Amgen. Male *db*/*db* mice on a BKS background were delivered at 8 weeks of age (Jackson Laboratory, 000642). Upon arrival, mice were group-housed at four animals per cage and the cages were changed at least twice per week. Animals had ad libitum access to irradiated pelleted feed (Envigo Teklad Global Soy Protein-free Extruded Rodent Diet, 2020X). Animals were acclimated to manual hand restraint beginning at 5 d before the study. BW and blood glucose were measured as a pre-screen to exclude mice from the study with low BW or blood glucose levels. On the day of the study, baseline BW and blood glucose were measured. Using the cage mean for blood glucose and BW, the mice were assigned to vehicle or treatment groups by cage with equal distribution of blood glucose and BW. Vehicle or AMG 133 murine surrogate (2.0 mg kg^−1^) was subsequently i.p. injected. BW was measured before injection (0 h) and at 24-h intervals thereafter up to 216 h after injection. Blood glucose was measured before injection (0 h), at 4 h and 24 h and at 24-h intervals thereafter up to 144 h after the injection under ad libitum feeding conditions. Blood samples were taken from the retro-orbital sinus vein from each conscious mouse and blood glucose was measured using an AlphaTrak glucometer (AlphaTrak 2, Zoetis).

### DIO mice

The DIO mouse study was conducted at Amgen. Male C57BL/6N mice were delivered at approximately 4 weeks of age from Envigo (formerly Harlan Laboratories). Animals were group-housed (two to four mice per cage) and following 1 week of acclimation, were fed a high-fat diet (HFD) containing 60% of energy from fat enriched with saturated fatty acids (D12492, Research Diets) for an additional 8 weeks. Mice were then single-housed and continued with the HFD feeding for the remainder of the study. Following a total of 17 weeks of HFD feeding, mice were acclimated to manual hand restraint and baseline blood glucose levels were measured (day −3). Mice were assigned into vehicle and treatment groups using blood glucose (day −3) and BW (day 0) values as stratification criteria, with equal distribution of blood glucose and BW. Vehicle, a low-dose AMG 133 murine surrogate (0.5 mg kg^−1^) or a high-dose AMG 133 murine surrogate (2.5 mg kg^−1^) was i.p. injected into 22-week-old male DIO mice every 6 d on study days 0, 6 and 12. The last dose was administered on study day 12 and the study was terminated on study day 18. All data from two animals in the high-dose group were excluded following the conclusion of the study due to excessive BW loss. BW loss for these two animals exceeded 45%, whereas all remaining animals in the high-dose group lost less than 36%. Due to the consistency of the HFD and the tendency to crumble at ambient temperature, reliable food intake measurements could not be recorded for certain mice in each group on certain days. Data are represented as *n* = 5–8 per group. Due to limited plasma volume for one mouse in vehicle and one mouse in the AMG 133 murine surrogate 2.5 mg kg^−1^ group, data are represented as *n* = 5–7 for triglycerides and cholesterol.

### Obese cynomolgus monkeys

Obese cynomolgus monkeys (*M.* *fascicularis*, male, BW > 7 kg; BMI > 41 kg m^−^^2^) were obtained from KBI’s stock colony and were individually housed in stainless-steel cages for the duration of the study. As described previously, ambient temperature and humidity range were set at 64–84 °F and 30–70%, respectively and lighting in the animal-holding rooms was maintained on a 12-h light–dark cycle^34^. Monkeys had continuous access to clean water, were fed standard chow (proprietary to KBI) twice daily and received an apple snack once daily. The monkeys were provided with environmental enrichment.

At least 95 naive male obese cynomolgus monkeys with latest BMI > 41 kg m^−2^ were pre-selected from the monkey colony. A total of 85 monkeys were selected to undergo training based on veterinary assessment. After several weeks of training for all study-related procedures, 70 out of 85 monkeys were selected based on blood chemistry and BW data collected at the end of the training period. The monkeys were assigned to seven groups (*n* = 10 monkeys per group) with equal distribution of blood chemistries and BW. Data from only three groups (vehicle, AMG 133 0.25 mg kg^−1^ and AMG 133 0.75 mg kg^−1^) are reported and discussed in this publication. Once-weekly dosing by s.c. injection occurred for 6 weeks followed by a 3-week washout period. BW, blood chemistries and metabolic profiling were measured weekly; food and water intake were monitored daily. One monkey from the AMG 133 0.25 mg kg^−1^ group and two monkeys from the AMG 133 0.75 mg kg^−1^ group were excluded from the study/data analysis due to loss of exposure (antidrug antibody-mediated clearance).

### Statistical analysis for animal studies

Statistical analyses were performed using GraphPad Prism v.8.4.3. Descriptive statistics (mean ± s.e.m.) were used to summarize the results. Statistical significance was defined as *P < 0.05, **P < 0.01, ***P < 0.001 and ****P < 0.0001 for vehicle versus AMG 133 murine surrogate or AMG 133-treated groups; or AMG 133 murine surrogate or AMG 133-treated groups compared to each other (Figs. 2a–h and 3a,c–f) and ^++^*P* < 0.01, ^+++^*P* < 0.001 for AMG 133 murine surrogate-treated groups compared to each dose level (Fig. 2c) or ^+^*P* < 0.05 for AMG 133 treated groups compared to each dose level (Fig. 3a) using one-way or two-way ANOVA or mixed-effects analysis with Tukey’s or Sidak’s multiple comparison analysis.

### Phase 1 clinical study

#### Clinical study design

This was an FIH, double-blind, randomized, placebo-controlled study consisting of two parts: SAD and MAD in participants with obesity (NCT04478708). Three clinical sites in the United States conducted this trial, starting in August 2020 and completing in November 2022. This paper reports final analysis data from the five SAD and three MAD cohorts (75 participants total) with a date of January 2023. A single-dose i.v. cohort with eight participants was included in the study to determine bioavailability but was not reported in the current dataset. In addition, one exploratory cohort that enroled 13 participants was used to evaluate digital tools and two open-label cohorts enroled 14 participants to evaluate dose-escalation strategies. These additional multiple-dose cohorts were not included in the current dataset. These exploratory cohorts included dose levels reported in the SAD and MAD cohorts. The CONSORT diagram reporting disposition for all 110 enroled participants is shown in Extended Data Fig. 3. Sex was based on the birth gender/sex of the participants. Biological parameters may be different between males/men and females/women and have been determined by studying the population according to the specific to gender/sex assigned at birth rather than self-reported gender.

Eligible participants were women of nonreproductive potential and men aged 18–65 years with a BMI between 30.0 kg m^−2^ and 40.0 kg m^−2^ and HbA1c ≤ 6.5% and/or a fasting glucose of <125 mg dl^−1^. Participants had normal vital signs, 12-lead electrocardiogram results and clinical laboratory tests at the time of randomization.

Participants were randomly assigned 3:1 to receive AMG 133 or placebo. The SAD evaluated AMG 133 doses ranging from 21 mg to 840 mg in six cohorts. The MAD evaluated doses ranging from 140 mg to 420 mg administered every 4 weeks on days 1, 29 and 57. The sentinel pair was observed for at least 48 h before the remaining participants were dosed, provided there were no safety or tolerability concerns as assessed by the investigator. Enrolment into the SAD and MAD cohorts was sequential. Subsequent cohorts were dosed after the dosing regimen in the preceding cohort was recommended to be safe and well tolerated based on the safety and laboratory data through at least day 15. Participants received AMG 133 in vials containing 70 mg ml^−1^ of AMG 133. Placebo was provided as normal saline, the volume matching the investigational drug at each dose level. AMG 133 or matching placebo was administered in the morning (fasting) by s.c. injection in the abdomen of the participant.

#### Clinical outcomes

The primary end points were the participant incidence of TEAEs, changes in laboratory safety tests, vital signs and 12-lead electrocardiograms. The assessment of TEAE severity was based on the Amgen standard grading scale as follows: mild (aware of symptom, but easily tolerated), moderate (discomfort enough to cause interference with usual activity) and severe (incapacitating with inability to do work or do usual activity). The secondary end points were AMG 133 PK parameters, including *t*_max_ and *t*_1/2_. The exploratory end points were PD parameters, including concentrations of fasting glucose, insulin, C-peptide, glucagon, free fatty acid, lipids, HbA1c and changes in BW, waist circumference and BMI.

#### PK and PD analysis

The PK of AMG 133 was evaluated using a validated liquid-chromatography tandem mass spectrometry (LC–MS/MS) methodology to detect total AMG 133, defined as the GIPR-Ab with or without the GLP-1 analogue peptides and intact AMG 133, defined as the GIPR-Ab with at least one attached GLP-1 analogue peptide. The lower limit of quantification for the analytical assay was 50 ng ml^−1^. PK parameters were analysed using standard noncompartmental analysis in Phoenix WinNonlin.

#### Statistical analysis for phase 1 clinical study

The sample size of up to eight participants per cohort for this SAD and MAD trial was based on conventional study designs for FIH exploratory trials to obtain adequate safety, tolerability and PK data. Considering the study was not based on any statistical inferences, all PK, PD and safety parameters were analysed descriptively and no formal hypothesis test-driven sample size calculations were performed. Continuous data were summarized using mean and s.d. or s.e.m., whereas categorical data were summarized using frequency counts and percentages. Post hoc *t*-tests were performed to provide a measure toward proof of concept for various parameters at the end of dosing where the data distribution was assumed to be normal but this was not formally tested (Figs. 4 and 5, Extended Data Fig. 4 and Extended Data Table 3). The full analysis set consisted of all randomized participants who receive at least one dose of AMG 133. The safety analysis set was the same as the full analysis set. The PK analysis set consisted of all participants who received at least one dose of AMG 133 for whom at least one PK parameter or end point could be adequately estimated. No missing data were imputed. All analyses were performed with the use of SAS software, v.9.4 (SAS Institute) and GraphPad Prism v.8.4.3.

### Reporting summary

Further information on research design is available in the Nature Portfolio Reporting Summary linked to this article.

## Supplementary information

Supplementary Information Process development and manufacturing details.

Reporting Summary

CONSORT checklist.

## Source data

Source Data Fig. 2 Statistical source data.

Source Data Fig. 3 Statistical source data.

Source Data Fig. 4 Statistical source data.

Source Data Fig. 5 Statistical source data.

Source Data Extended Data Fig. 2 Statistical source data.

Source Data Extended Data Fig. 4 Statistical source data.

Source Data Extended Data Fig. 5 Statistical source data.

## Figures and Tables

**Extended Data Table 1:**
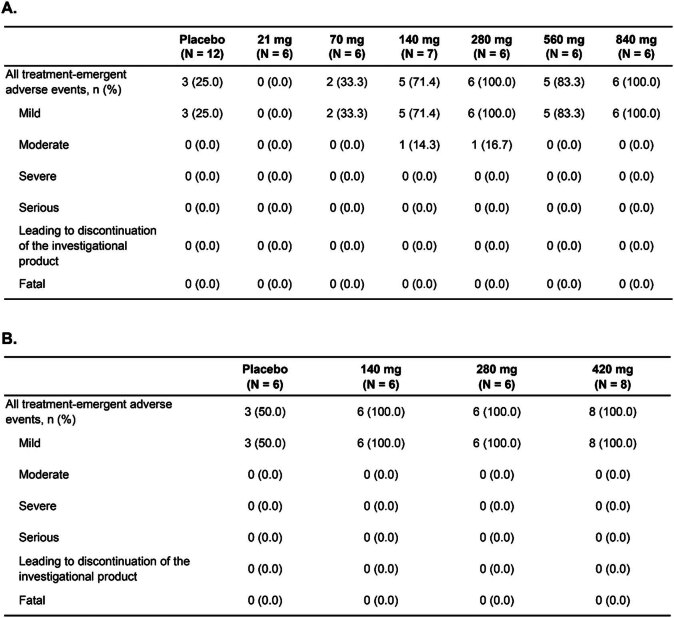
Treatment-emergent adverse events in humans after a single ascending dose (A) or multiple ascending doses (B) of placebo and AMG 133

Data presented as n (%). N, total number of participants within each group.

**Extended Data Table 2:**
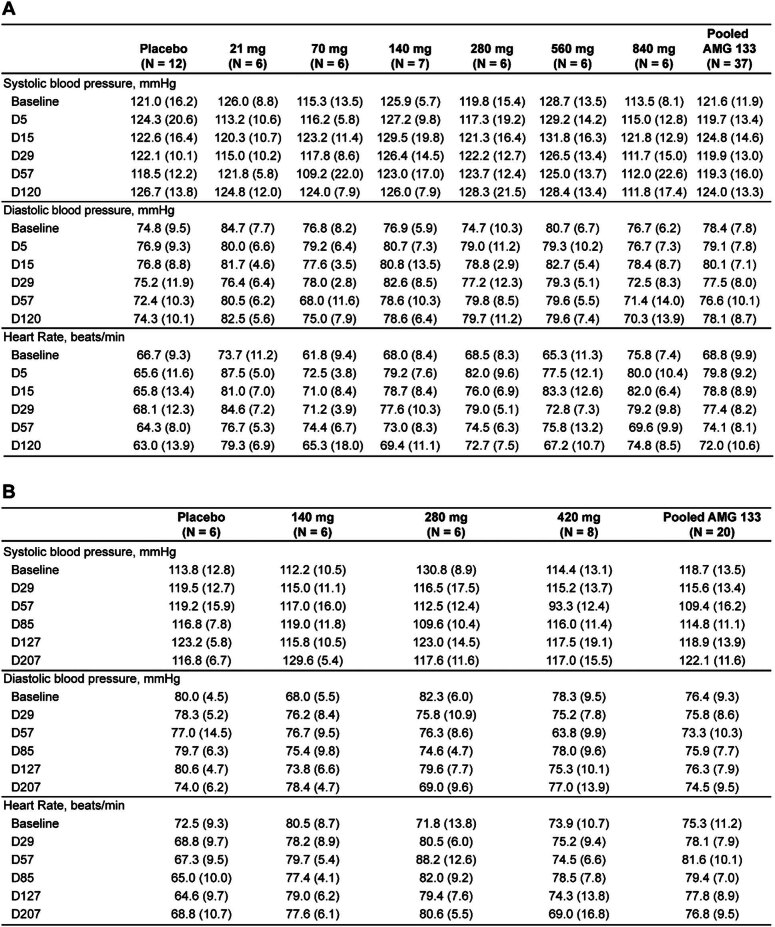
Vital sign measures in humans after a single ascending dose (A) or multiple ascending doses (B) of placebo and AMG 133

Data presented as mean (SD). N, total number of participants within each group.

**Extended Data Table 3:**
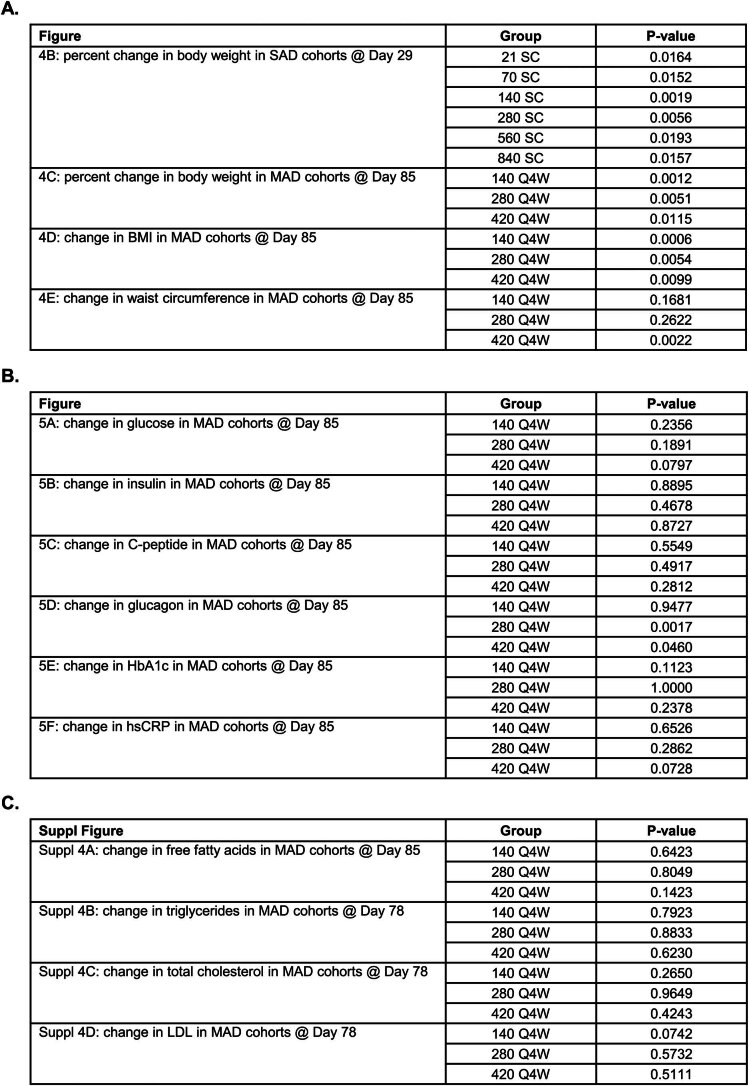
Post-hoc *t*-test results for clinical results including pharmacodynamic biomarkers (A), metabolic and inflammatory parameters (B), and free fatty acids and lipids (C)

Welch two-sided *t*-test was used with no multiplicity adjustments.

## Data Availability

The clinical study was conducted at three phase 1 clinical sites in the United States (Orange County Research Center, Anaheim Clinical Trials and Clinical Pharmacology of Miami) starting from August 2020 and completing in November 2022. The redacted study protocols and statistical analysis plan are provided as part of the manuscript files. Source data have also been published for the Figures and Extended Data Figures as per journal policy. There is a plan to share other data. This may include de-identified individual patient data for variables necessary to address the specific research question in an approved data-sharing request; also related data dictionaries, informed consent form and/or clinical study report. Data-sharing requests relating to data in this manuscript will be considered after the publication date and either (1) when this product and indication (or other new use) have been granted marketing authorization in both the US and Europe, or (2) when clinical development discontinues and the data will not be submitted to regulatory authorities. There is no end date for eligibility to submit a data-sharing request for these data. Qualified researchers may submit a request containing the research objectives, the Amgen product(s) and Amgen study/studies in scope, end points/outcomes of interest, statistical analysis plan, data requirements, publication plan and qualifications of the researcher(s). In general, Amgen does not grant external requests for individual patient data for the purpose of re-evaluating safety and efficacy issues already addressed in product labelling. A committee of internal advisors reviews requests. If not approved, requests may be further arbitrated by a Data-Sharing Independent Review Panel. Requests that pose a potential conflict of interest or an actual or potential competitive risk may be declined at Amgen’s sole discretion and without further arbitration. Upon approval, information necessary to address the research question will be provided under the terms of a data-sharing agreement. This may include anonymized individual patient data and/or available supporting documents, containing fragments of analysis code provided in analysis specifications. Inquiries specific to this manuscript may be made to M.M.V. (mveniant@amgen.com) and further details regarding general data availability matters are available at https://wwwext.amgen.com/science/clinical-trials/clinical-data-transparency-practices/. Source data are provided with this paper.
