# Supplementary material for: A GIPR antagonist conjugated to GLP-1 analogues promotes weight loss with improved metabolic parameters in preclinical and phase 1 settings
Source: Nat Metab. 2024 Feb 5;6(2):290–303. doi: 10.1038/s42255-023-00966-w (PMC10896721; doi:10.1038/s42255-023-00966-w)
Supplement: Supplementary file 1 — Process development and manufacturing details. [file 42255_2023_966_MOESM1_ESM.pdf]

nature metabolism

Article

https://doi.org/10.1038/s42255-023-00966-w

A GIPR antagonist conjugated to GLP-1 analogues promotes weight loss with improved metabolic parameters in preclinical and phase 1 settings

In the format provided by the authors and unedited

Process Development and Manufacturing

A preliminary manufacturability assessment was conducted to identify the lead Ab and peptide candidates. The peptide and Ab were advanced for process development and manufacturing of the phase 1 material. Process development activities included selecting the lead Ab with high expression and favorable product characteristics, developing the conjugation process to achieve robust conjugation efficiency, and assessing stability of the drug substance as well as the drug product. The product attributes were tested and monitored using analytical assays.

The drug substance and drug product processes started from a vial with cells that were thawed and expanded till the production bioreactor. The expressed anti-GIPR protein from the production bioreactor was harvested, purified, and conjugated to the GLP-analog peptide to manufacture drug substance. Drug substance was then filled into drug product using Amgen's drug product manufacturing process. The large-scale manufacturing campaigns conducted for phase 1 supply included a drug supply for toxicology studies conducted at 500-liter bioreactor scale and a clinical drug supply conducted at 2,000-liter bioreactor scale. The material generated from toxicology and clinical drug supply showed analytical comparability. Additionally, stability studies conducted to date show the product attributes to be within the specification limits.

Further process development activities are in progress to design a commercial manufacturing process.
