# Supplementary material for: A GIPR antagonist conjugated to GLP-1 analogues promotes weight loss with improved metabolic parameters in preclinical and phase 1 settings
Source: Nat Metab. 2024 Feb 5;6(2):290–303. doi: 10.1038/s42255-023-00966-w (PMC10896721; doi:10.1038/s42255-023-00966-w)
Supplement: Supplementary file 2 — Reporting Summary [file 42255_2023_966_MOESM2_ESM.pdf]

nature portfolio

Corresponding author(s): Murielle Veniant Ellison

Last updated by author(s): Dec 1, 2023

Reporting Summary

Nature Portfolio wishes to improve the reproducibility of the work that we publish. This form provides structure for consistency and transparency in reporting. For further information on Nature Portfolio policies, see our Editorial Policies and the Editorial Policy Checklist.

Statistics

For all statistical analyses, confirm that the following items are present in the figure legend, table legend, main text, or Methods section.

n/a	Confirmed	
☐	☒	The exact sample size (*n*) for each experimental group/condition, given as a discrete number and unit of measurement
☐	☒	A statement on whether measurements were taken from distinct samples or whether the same sample was measured repeatedly
☐	☒	The statistical test(s) used AND whether they are one- or two-sided<br>*Only common tests should be described solely by name; describe more complex techniques in the Methods section.*
☒	☐	A description of all covariates tested
☒	☐	A description of any assumptions or corrections, such as tests of normality and adjustment for multiple comparisons
☐	☒	A full description of the statistical parameters including central tendency (e.g. means) or other basic estimates (e.g. regression coefficient) AND variation (e.g. standard deviation) or associated estimates of uncertainty (e.g. confidence intervals)
☐	☒	For null hypothesis testing, the test statistic (e.g. *F*, *t*, *r*) with confidence intervals, effect sizes, degrees of freedom and *P* value noted<br>*Give P values as exact values whenever suitable.*
☒	☐	For Bayesian analysis, information on the choice of priors and Markov chain Monte Carlo settings
☒	☐	For hierarchical and complex designs, identification of the appropriate level for tests and full reporting of outcomes
☒	☐	Estimates of effect sizes (e.g. Cohen's *d*, Pearson's *r*), indicating how they were calculated

Our web collection on statistics for biologists contains articles on many of the points above.

Software and code

Policy information about availability of computer code

Data collection	No code was used
Data analysis	Clinical data were analyzed using SAS software, version 9.4 (SAS Institute, Cary, NC) and GraphPad Prism 8.4.3. Watson LIMS (v7.4; Thermo Fisher) was used for bioanalytical concentration data analysis. Noncompartmental analysis was performed for PK parameter estimation using Phoenix® WinNonlin® (Certara, Princeton, NJ).

For manuscripts utilizing custom algorithms or software that are central to the research but not yet described in published literature, software must be made available to editors and reviewers. We strongly encourage code deposition in a community repository (e.g. GitHub). See the Nature Portfolio guidelines for submitting code & software for further information.

Data

Policy information about availability of data

All manuscripts must include a data availability statement. This statement should provide the following information, where applicable:

- Accession codes, unique identifiers, or web links for publicly available datasets
- A description of any restrictions on data availability
- For clinical datasets or third party data, please ensure that the statement adheres to our policy

Qualified researchers may request data from Amgen clinical studies. Complete details are available at the following: https://wwwext.amgen.com/science/clinical-trials/clinical-data-transparency-practices/

Human research participants

Policy information about studies involving human research participants and Sex and Gender in Research.

Reporting on sex and gender	In the first in human trial, the sex of the participants was reported as a number and percentage of the total number of participants. The sex and birth gender was based on self-reporting.
Population characteristics	Female of non-reproductive potential and male participants were included with age between 18 years to 65 years and a BMI between 30.0 kg/m2 and 40.0 kg/m2. Other than obesity, the participants were otherwise healthy as determined by the investigator or medically qualified designee based on a medical evaluation including medical history, physical examination, laboratory tests, and ECGs. Female participants were excluded from the study if they were of child bearing potential due to the unknown safety risks of the AMG 133 in pregnancy.
Recruitment	The participants were recruited from the clinical sites using a combination of internal database searching and advertising as needed. In keeping with industry standards, the clinical sites were selected for the study based on feasibility assessments to ensure prior experience and regulatory compliance in the conduct of metabolic trials and access to the participant population of interest. The participant selection was based on strict inclusion/exclusion criteria that were prespecified in the protocol; therefore, selection biases that may affect the data were minimized and the intented population was included in the trial.
Ethics oversight	The protocol and informed consent form were reviewed and approved by the Institutional Review Boards at the clinical sites, including Orange County Research Center (Tustin, CA 92780), Anaheim Clinical Trials (Anaheim, CA 92801), and Clinical Pharmacology of Miami, LLC (Miami, FL 33014).

Note that full information on the approval of the study protocol must also be provided in the manuscript.

Field-specific reporting

Please select the one below that is the best fit for your research. If you are not sure, read the appropriate sections before making your selection.

☒ Life sciences ☐ Behavioural & social sciences ☐ Ecological, evolutionary & environmental sciences

For a reference copy of the document with all sections, see nature.com/documents/nr-reporting-summary-flat.pdf

Life sciences study design

All studies must disclose on these points even when the disclosure is negative.

Sample size	Clinical study: The sample size was not based on any statistical inferences. The sample size is typical for Ph1 studies with at least 6 participants receiving AMG 133 in each cohort to evaluate safety, pharmacokinetic, and pharmacodynamic parameters and was considered sufficient to evaluate the primary outcome of this study which was safety and tolerability. The sample size was sufficiently powered to detect common adverse events. Approximately 112 subjects were planned to be enrolled in the study, for which there is at least 92.9% chance of detecting an adverse event with a true incidence rate of 3% or greater and at least 98.8% chance of detecting an adverse event with a true incidence rate of 5% or greater.<br>Preclinical studies: No statistical methods were used to pre-determine sample sizes for preclinical experiments, but our sample sizes are similar to those reported in previous publications (PMID: 34095876) and based on internal historical studies/data. In addition, it was confirmed that there was no statistical difference in any sorting parameter prior to starting treatment.
Data exclusions	No data excluded from analysis; manuscript reports data from SAD/MAD cohorts; doesn't include data from 4 additional exploratory cohorts. For preclinical studies, certain animals or data points are excluded with specifically stated reasons: 2 DIO mice excluded from study and data analysis due to outlier BW response, or lack of plasma volume for plasma chemistry measurements (2 data points) or unreliable food intake data (several data points); 3 monkeys are excluded due to loss of drug exposure due to anti-drug-antibodies
Replication	Clinical study: The study was run in several participant cohorts, allowing internal replication of results across dose groups. The clinical samples were analyzed for each analyte one time per sample.<br>In vitro cAMP assays: all data are reproducible in n>3 independent experiments.<br>Preclinical studies (dbs, DIOs, obese cynos) each single independent studies
Randomization	Clinical study: For the phase 1 study, the participants were randomly assigned to treatment goups.<br>In vitro cAMP assays: not applicable<br>Preclinical studies (dbs, DIOs, obese cynos): animals were manually sorted so that there was no statistically significant differences in sorting parameters prior to study start; no program was used to determine group size, this was based on internal historical studies/data.
Blinding	Clinical study: This was a double blind study. Participants and investigators were blinded to treatment allocation during dosing, data collection and analysis.<br>In vitro cAMP assays: Investigators were not blinded.<br>Preclinical studies (dbs, DIOs, obese cynos): db and DIO studies were not blinded; obese cyno study was blinded

Reporting for specific materials, systems and methods

We require information from authors about some types of materials, experimental systems and methods used in many studies. Here, indicate whether each material, system or method listed is relevant to your study. If you are not sure if a list item applies to your research, read the appropriate section before selecting a response.

Materials & experimental systems

n/a	Involved in the study
☐	☒ Antibodies
☐	☒ Eukaryotic cell lines
☒	☐ Palaeontology and archaeology
☐	☒ Animals and other organisms
☐	☒ Clinical data
☒	☐ Dual use research of concern

Methods

n/a	Involved in the study
☒	☐ ChIP-seq
☒	☐ Flow cytometry
☒	☐ MRI-based neuroimaging

Antibodies

Antibodies used

Preclinical PK assays utilized the following monoclonal antibodies: mouse anti-human GLP-1 (Thermo Fisher Scientific, Cat#ABS 033-04-02; Clone 4), mouse anti-human IgG Fc (Amgen, Clone 1.35), and mouse anti-human IgG Fc (Amgen, Clone 21.1). Preclinical PK was assessed using ELISA; clinical PK was measured using LC-MS/MS.

Validation

Validation of commercial anti-GLP-1 antibody (Clone 4) is provided in manufacturer product information. As stated on the manufacturer's website, "ABS 033-04-02 binds the free N-terminus of GLP-1(7-37) and GLP-1(7-36) amide and shows < 0.2% cross-reactivity with GLP-1(1-37), GLP-1(9-36) amide, glucagon, human GIP and exendin-4. ABS 033-04-02 cross-reacts approximately 1% with human GLP-2." Additionally, in routine assay verification procedures utilizing the sandwich ELISA application described for intact AMG 133 bioanalysis (anti-IgG Fc antibody Clone 1.35 paired with anti-GLP1 antibody Clone 4), detection of AMG 133 in preclinical matrices was specific, sensitive, reproducible, and fit-for-purpose.

Validation of Amgen proprietary anti-IgG Fc antibodies (Clones 1.35 and 21.1) is described in a publication, "Implementation of a universal analytical method in early-stage development of human antibody therapeutics: application to pharmacokinetic assessment for candidate selection", Shih et al, Bioanalysis, 2012, 4(19), 2357–2365. Additionally, in routine assay verification procedures utilizing the sandwich ELISA application described for total AMG 133 bioanalysis (anti-IgG Fc antibody Clone 1.35 paired with anti-IgG Fc antibody Clone 21.1), detection of AMG 133 in preclinical matrices was specific, sensitive, reproducible, and fit-for-purpose.

Eukaryotic cell lines

Policy information about cell lines and Sex and Gender in Research

Cell line source(s)

Parental CHO-K1 and HEK 293T cells were obtained from ATCC. CHO cells stably expressing human, mouse, rat and monkey GLP-1R, HEK 293T cells stably expressing human or monkey GIPR and CHO-AM1D stably expressing mouse or rat GIPR were all generated at Amgen Inc.

Authentication

Cell lines were not independently authenticated

Mycoplasma contamination

All cell lines tested negative for mycoplasma contamination

Commonly misidentified lines
(See ICLAC register)

None

Animals and other research organisms

Policy information about studies involving animals; ARRIVE guidelines recommended for reporting animal research, and Sex and Gender in Research

Laboratory animals

Mice and monkeys were housed at AAALAC International accredited facilities and were maintained in accordance with IACUC oversight.

Mice (Mus musculus) were housed in individual static or ventilated caging (IVC) system (Tecniplast) on an irradiated corncob bedding (Envigo Teklad 7097). Lighting in animal holding rooms was maintained on 12:12 hr. light: dark cycle, and the ambient temperature and humidity range was at 68 to 79 F and 30 to 70%, respectively. Animals had ad libitum access to irradiated pelleted feed (Research Diets Inc irradiated D12492 60% kcal high fat diet or Envigo Teklad Global Rodent Diet-soy protein free extruded, irradiated 2029X) and reverse-osmosis (RO) chlorinated (2 to 3 ppm) water via an automatic watering system. Male diet induced obese (DIO) C57Bl/6 mice (Envigo), 22 weeks of age; male db/db mice (Jackson Laboratory stock #000642; BKS.Cg-Dock7m +/+ Leprdb/J), 9 weeks of age, CD-1 IGS mice (Crl:CD1(ICR))(Charles River Laboratories); 8-12 weeks of age.

Monkeys (Macaca fascicularis), naïve, spontaneously obese, 9-14 years of age, were obtained from KBI stock colony and evaluated by

	veterinarian prior to study enrollment. The animals were individually housed in stainless steel cages for the duration of the study under a controlled environment that was set to maintain a temperature of 18-29°C and a minimum of 10 air changes/hour. Relative humidity was naturally within the range of 30-70%. A time-controlled lighting system was used to provide a regular 12-hour light/12-hour dark diurnal cycle (light 7:00 AM-7:00 PM). The records of temperature and humidity measurements obtained for the duration of the in-life phase were maintained in facility records. Monkeys were fed morning and evening meal (unlimited KBI proprietary standard monkey formula feed (extruded pellets) in 1hr period), plus apple snack (150g) in afternoon. Water was provided via a 500-mL water bottle. Each animal was provided with up to 5000 mL of water per day. Availability of drinking water was monitored at all times. All water bottles were filled prior to start of the 12-hour dark diurnal cycle. Certificate of analysis of diet and water analysis results were maintained in facility records.
Wild animals	No wild animals were used in these studies.
Reporting on sex	PK studies were performed in female monkeys; all other studies were performed in male mice or monkeys.
Field-collected samples	No field-collected samples were used in these studies.
Ethics oversight	All studies with non-human animals complied with relevant ethical regulations and were approved by Amgen Institutional Animal Care and Use Committee (IACUC), KBI Institutional Animal Care and Use Committee (IACUC), Amgen External Study Ethical Review Committee (ESERC).

Note that full information on the approval of the study protocol must also be provided in the manuscript.

Clinical data

Policy information about clinical studies

All manuscripts should comply with the ICMJE guidelines for publication of clinical research and a completed CONSORT checklist must be included with all submissions.

Clinical trial registration	NCT04478708
Study protocol	The study completed November 18 2022. This paper is reporting final analysis data from the SAD and MAD cohorts. There are an additional 4 exploratory cohorts not reported in this manuscript. The redacted study protocol is provided with the manuscript.
Data collection	The clinical study was conducted at 3 Phase 1 clinical sites in the US (Orange County Research Center [Tustin, CA 92780], Anaheim Clinical Trials [Anaheim, CA 92801], and Clinical Pharmacology of Miami, LLC [Miami, FL 33014]) starting from August 2020 and completing in November 2022. The data were collected by trained study staff at each research facility according to a detailed study protocol and with the oversight of the sponsor to ensure good clinical practices and adherence to the protocol. Subjects were randomized 3:1 to be dosed with AMG 133 or placebo.
Outcomes	For all endpoints, methods for assessments were standard for clinical studies and were predefined in the approved study protocol, with a schedule timing for when each assessment was to occur. All study sites were trained on the protocol and how to conduct assessments. The primary endpoints were the participant incidence of TEAEs, changes in laboratory safety tests, vital signs, and 12-lead electrocardiograms. The secondary endpoints were AMG 133 PK parameters including, but not limited to, Cmax during a dosing interval, the time of maximum observed concentration (Tmax), and AUC. The exploratory endpoints were PD parameters including, but not limited to, concentrations of fasting glucose, insulin, C-peptide, glucagon, free fatty acid, lipids, HbA1c, GIP and GLP-1, and changes in BW, waist circumference and BMI.
